# Methods for mitochondrial health assessment by High Content Imaging System

**DOI:** 10.1016/j.mex.2022.101685

**Published:** 2022-04-02

**Authors:** Chatnapa Panusatid, Nattachai Thangsiriskul, Chayanon Peerapittayamongkol

**Affiliations:** Department of Biochemistry, Faculty of Medicine Siriraj Hospital, Mahidol University, Bangkok, 10700, Thailand

**Keywords:** Mitochondrial function, Mitochondrial ROS, Mitochondrial membrane potential, Mitochondrial morphology, High content fluorescent imaging

## Abstract

Mitochondria are important organelles responsible for energy production. Mitochondrial dysfunction relates to various pathological diseases. The investigation of mitochondrial heath is critical to evaluate the cellular status. Herein, we demonstrated an approach for determining the status of mitochondrial health by observing mitochondrial H_2_O_2_ (one type of ROS), membrane potential, and morphology (fragmentation and length) in live primary fibroblast cells. The cells were co-stained with fluorescent dyes (Hoechst 33342 and MITO-ID® Red/MitoPY1/JC-10) and continuously processed by the High Content Imaging System. We employed the Operetta CLS^TM^ to take fluorescent images with its given quickness and high resolution. The CellProfiler image analysis software was further used to identify cell and mitochondrial phenotypes in the thousand fluorescent images.

• We could quantitatively analyze fluorescent images with high-throughput and high-speed detection to track the alteration of mitochondrial status.
• The MMP assay is sensitive to FCCP even at the concentration of 0.01 µM.
• The fibroblast cells treated with stress inducers (H_2_O_2_, FCCP, and phenanthroline) revealed a significant change in mitochondrial health parameters, with more ROS accumulation, depolarized MMP, increased fragmentation, and reduced length of mitochondria.

## Graphica abstract

**Figure:**
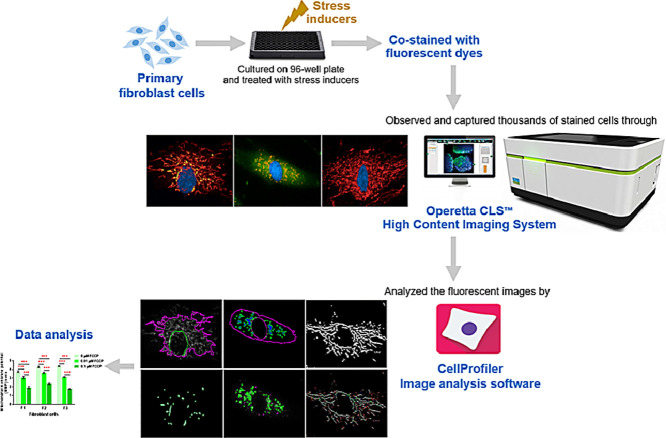

Specifications table

**Table:** 

Subject Area:	Biochemistry, Genetics and Molecular Biology
More specific subject area:	Mitochondria - Fluorescent imaging analysis
Method name:	Mitochondrial fluorescent staining - High Content Imaging System
Name and reference of original method:	Carpenter, A.E., et al., *CellProfiler: image analysis software for identifying and quantifying cell phenotypes.* Genome Biol, 2006. 7(10): p. R100. Dickinson, B.C., V.S. Lin, and C.J. Chang, *Preparation and use of MitoPY1 for imaging hydrogen peroxide in mitochondria of live cells.* Nat Protoc, 2013. 8(6): p. 1249-59. Sakamuru, S., M.S. Attene-Ramos, and M. Xia, *Mitochondrial Membrane Potential Assay.* Methods Mol Biol, 2016. 1473: p. 17-22. Park, S.J., et al., *Mitochondrial fragmentation caused by phenanthroline promotes mitophagy.* FEBS Lett, 2012. 586(24): p. 4303-10.
Resource availability:	https://www.perkinelmer.com/th/category/operetta-cls-high-content-analysis-system https://cellprofiler.org/

## Introduction

Mitochondria are powerful organelles involved in various vital cellular functions. The key role is to maintain cellular homeostasis and energy status by producing ATP. ATP is generated via oxidative phosphorylation through the electron transport chain (ETC) in the inner membrane of mitochondria. The transferring of electrons through respiratory Complexes I, III, and IV creates an electrochemical gradient, a combination of mitochondrial membrane potential (MMP) and pH gradient. This electrochemical gradient provides free energy to drive ATP synthesis. During electron transports, reactive oxygen species (ROS) can occur from the leakage of electrons. The accumulation of ROS results in oxidative stress and mitochondrial damage. Therefore, the disruption of MMP and ROS accumulation further impairs the mitochondrial functions and often prevails cellular damage and apoptotic cell death pathway, eventually contributing to several diseases and aging [1, 2].

However, mitochondria have a vital defensive mechanism against mitochondrial damage by destroying impaired mitochondria and keeping a balance of healthy mitochondria. This dynamically balanced check is manifested by changes in mitochondrial morphology. The elongation of mitochondria, tubular networks, is associated with mitochondrial health due to the fusion of mitochondria, which allows the exchange of healthy to damaged mitochondrial components to maintain the overall state in balance. On the other hand, mitochondrial fragmentation, the small pieces from the fission process, is related to an increase in ROS production and ATP depletion [3]. Excessive mitochondrial fission has been found to co-exist in cardiomyopathy [4], cancer, and neurodegenerative diseases such as Alzheimer's disease.

Therefore, mitochondrial health is a key for maintaining cellular stability and pertained to health or disease. In this study, we explored the throughput method for monitoring mitochondrial status, including mitochondrial ROS, membrane potential status, and morphology by using the High-Content Imaging System. We employed the Operetta CLS™ (PerkinElmer), a high-content fluorescent imaging technology with a high-throughput detection system that automates the capture of cell images from 96-well microplate. We selected a water immersion objective lens, which gives substantially brighter images compared to air objectives. CellProfiler image analysis, a free public software, was used to identify cell components, enabling us to generate and automatically analyze thousands of images through a pipeline for statistical analysis [5], [6], [7].

## Method details

### Cell culture

Primary fibroblast cells were established from skin biopsies of three volunteers (F1, F2, and F3). The Human Research Protection Unit approved this study with the certificate of approval No. Si 161/2019 (the Faculty of Medicine Siriraj Hospital, Mahidol University). The cells were grown in a T25 cell culture flask (Corning®) containing Dulbecco's modified Eagle's medium (DMEM, 5 mM glucose, Gibco®) supplemented with 10% fetal bovine serum (FBS), 1% penicillin (100 U/mL)/streptomycin (100 µg/mL), and 0.1% amphotericin B (1 mg/mL). Cells were incubated at 37°C with a humidified 5% CO_2_ atmosphere, and the culture medium was changed every other day.

### Treatment of stress inducers

Fibroblast cells at 80-90% confluency of a 25 mL flask were seeded on the 96-well plate (CellCarrier-96, PerkinElmer) with a density of 4,000-5,000 cells/well for 24 h. The next day, cells were treated with stress inducers including hydrogen peroxide (H_2_O_2_), carbonyl cyanide-*p*-trifluoromethoxyphenylhydrazone (FCCP), or phenanthroline with the final concentrations as indicated in Table 1 in the culture media for the measurement of mitochondrial ROS, MMP, and fragmentation and length, respectively.

**Table 1:** Preparation of H_2_O_2_, FCCP, and phenanthroline concentrations.

Stress inducers	Reconstituted solution	Stock solution	Final concentrations	Duration of treatment
H_2_O_2_ (Siribuncha Corp, Lot. No. 0230049)	Water	3% wt/vol (0.882 M)	100, 200, and 400 µM	1 h
FCCP (Sigma-Aldrich, Cat. No. C2920)	Absolute ethanol	5,000 µM	0.01 and 0.1 µM	1 h
Phenanthroline (Sigma-Aldrich, Cat.No.131377)	Absolute ethanol	25,000 µM	50 µM	4 h

### Cell staining

The stock solutions of fluorescent dyes were generated as shown in Table 2. Whereas MitoPY1 stock was prepared by dissolving in methanol, aliquoted and vacuum-evaporating the solvent [8]. In each experiment, MitoPY1 was freshly dissolved in DMSO. After treatment with stress inducers, the cells were washed twice with PBS. The final concentrations of mixture dyes prepared in the phenol-free DMEM medium supplemented with 10% FBS were added into the 96-well plate at 50 µL/well to stain the cells for 30-45 min in the dark. The cells were washed with PBS twice and replaced with phenol-free DMEM medium supplemented with 2% FBS to reduce the background noise before fluorescent imaging.

**Table 2:** Preparation of fluorescent dyes.

Fluorescent dyes	ReconstitutedSolution	Stock solution	Final concentrations	Duration of staining
Hoechst 33342 (Tocris Bioscience, Cat.No.5117)	Water	5 mM	3 µM	30-45 min
MITO-ID® Red (GFP-certified mitochondrial detection; Enzo Life Sciences, Cat.No.ENZ-51007)	DMSO	10,000-fold dilution of stock solution		30 min
MitoPY1 (Tocris Bioscience, Cat.No.4428)	DMSO	10 mM	10 µM	45 min
JC-10 (ultra-pure; Enzo Life Sciences, Cat.No.ENZ-52305)	DMSO	3 mM	10 µM	45 min

### High-content analysis system setting

The images of stained cells were visualized using the Operetta CLS^TM^ High-Content Analysis System (PerkinElmer) in the confocal or non-confocal with two peaks of autofocus mode and a water objective lens, 40x high numerical aperture (NA), which had a high refractive index. With its high resolution and consistent illumination from the 8x LED light source, the Operetta CLS could rapidly track changes in mitochondrial status to reduce photodamage at a rate of around one field per 2-4 seconds, depending on the percentage of power and the exposure time. We used a non-confocal mode for MMP measurement due to the JC-10 dye being particularly sensitive to the light source resulting in fluorescent signals being easily dropped. As a result, this mode is better suited for signal conservation. The other experiments, on the other hand, employ confocal mode. We captured images of thousands of stained cells on a 96-well plate (15–40 cells/field, 60–80 fields/well, and 2 wells/conditions) under 37°C and 5% CO_2_ control. The binning mode was set to 2. The images' final resolution was 0.299 µm pixel size, 16 bit per pixel, and 1080 × 1080. The Operetta CLS setting via the Harmony® 4.9 software was provided in Table 3.

**Table 3:** Setting of the Harmony® 4.9 software to control the Operetta CLS work in capturing fluorescent images.

Fluorescent channels	Mode	Exposure time (ms)	Power (%)	Excitation (nm)	Excitation filter (nm)	Emission (nm)	Emission filter (nm)
Hoechst 33342	Confocal Non-confocal	300 5	100 5	365	355-385	465	430-500
MITO-ID® Red	Confocal	1,300	100	550	530-560	680	655-705
MitoPY1	Confocal	1,500	100	510	490-515	553	525-580
JC-10 Rhodamine	Non-confocal	5	5	550	530-560	610	570-650
JC-10 Alexa 488	Non-confocal	5	10	475	460-490	525	500-550

### Image analysis procedure by the CellProfiler program

CellProfiler is a high-throughput cell image analysis tool that user can be used to build a pipeline to analyze the organelles of interest in cells [5], [6], [7]. We created the pipeline to identify and measure the number of nuclei, H_2_O_2_ in mitochondria, J-aggregate and J-monomers, and granularity and length of mitochondria from Hoechst 33342, MitoPY1, JC-10, and MITO-ID® Red fluorescent channels. The fluorescent images captured by the Operetta CLS were run through the pipeline in CellProfiler versions 3.1.9 or 4.0.7.

#### Metadata extraction (channel matching)

The fluorescent images from the Operetta CLS uploaded through the Columbus program were exported and extracted the metadata from the file name for specifying the image information and matching with the particular fluorescent channels using the regular expression; ^(?P<Row>[0-9]{3})(?P<Column>[0-9]{3})-(?P<Field>[0-9]{1,2})-(?P<Time>[0-9]{3})(?P<Stack>[0-9]{3})(?P<Channel>[0-9]{3}).tif. The original images from each channel were continuously selected as grayscale type i.e., each pixel representing a single intensity value.

#### Illumination correction

**Fig. 1:**
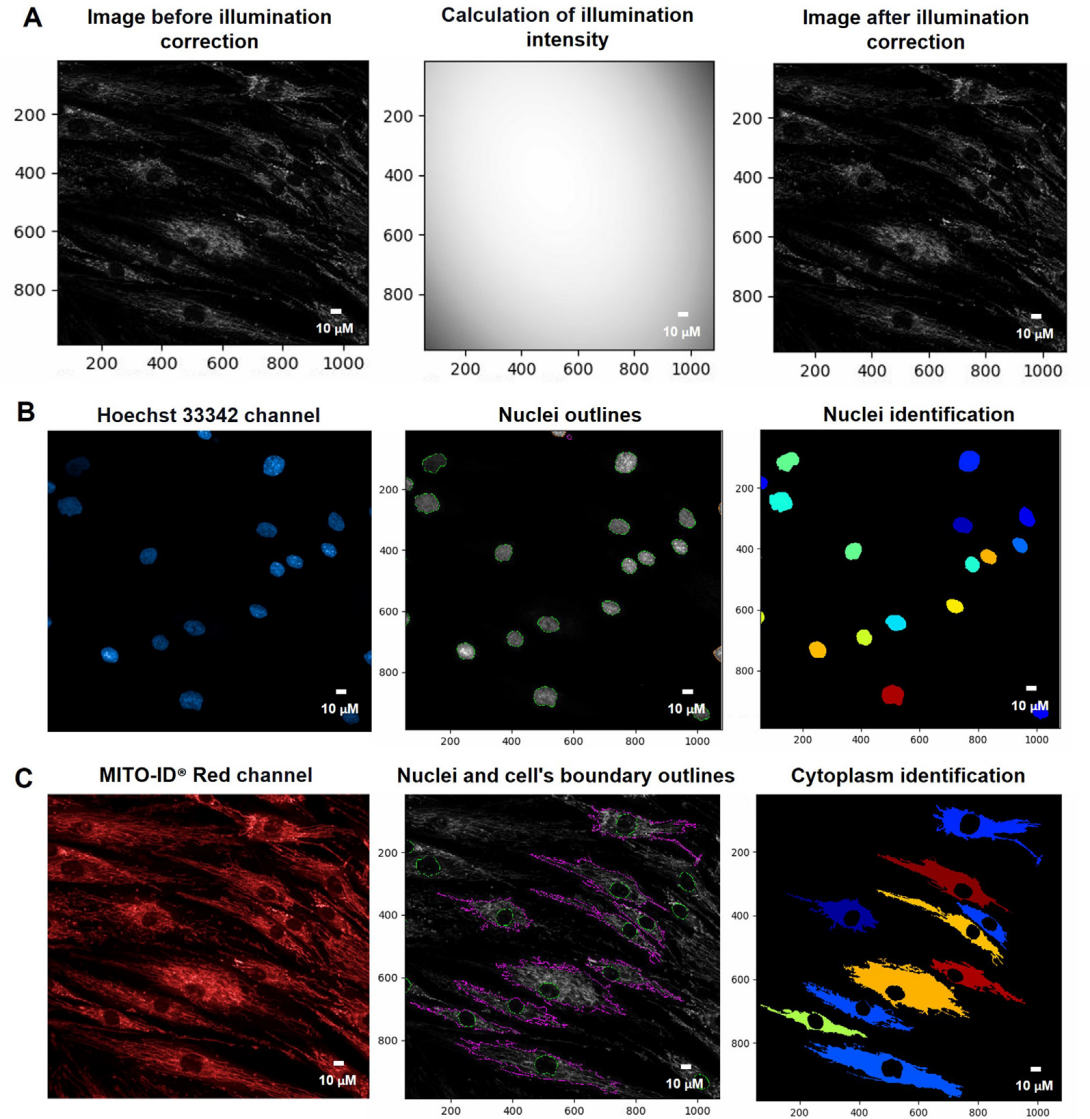
The pipeline for organelle segmentations by the CellProfiler program. A. An illumination correction was performed by using the Correction Illumination Calculate and Apply modules. B. The nuclei were determined from the Hoechst 33342 channel from the Identified Primary Object module. C. The cell's boundary and cytoplasm were singled out from the MITO-ID® Red channel using the Identify Secondary and Tertiary Object modules. Scale bars represent 10 µm. The pixel unit was used to scale the image.

One of the problems affecting precise fluorescence intensity measurements was that background illumination from the microscopy had an irregular pattern with bright or dark areas across the image. For reliable image analysis outcomes, we utilized the Correction Illumination Calculate and Apply modules: Fit Polynomial strategy to calculate the typical variance of illumination intensity and the Subtract strategy to apply the smoothed lightening pattern in each image (Fig. 1A) [9, 10].

#### Organelle segmentations

***Identify the primary object (nuclei)***

The primary object used to identify a cell's location was the stained nucleus with Hoechst 33342 dye. The threshold of fluorescent intensity of the Hoechst 33342 channel was determined by the Identified Primary Object modules: Global and Otsu thresholding methods defining the threshold into the foreground and background pixels by reducing the variation within each group. We further used the Three-classes thresholding strategy to classify fluorescent intensity above the threshold as foreground and below as background (Fig. 1B) [11].

#### Identify the secondary object (whole cell)

The secondary object, which was related to the primary object (the nuclei), was employed to determine a cell's boundary. We adopted the MITO-ID® Red channel to recognize the secondary object (Fig. 1C) for the mitochondrial H_2_O_2_, fragmentation, and length measurements. For the MMP measurement, the Alexa 488 channel was employed to determine J-monomer in the cytoplasm (Fig. 3A). The fluorescent intensity thresholds of the MITO-ID® Red and Alexa 488 channels were estimated by Global, Otsu, and classified intensity using the Three-classes Thresholding strategy. Similarly, the Propagation method was appointed as the determination of the dividing lines between the cells in contact.

#### Identify the tertiary object (cytoplasm)

**Fig. 2:**
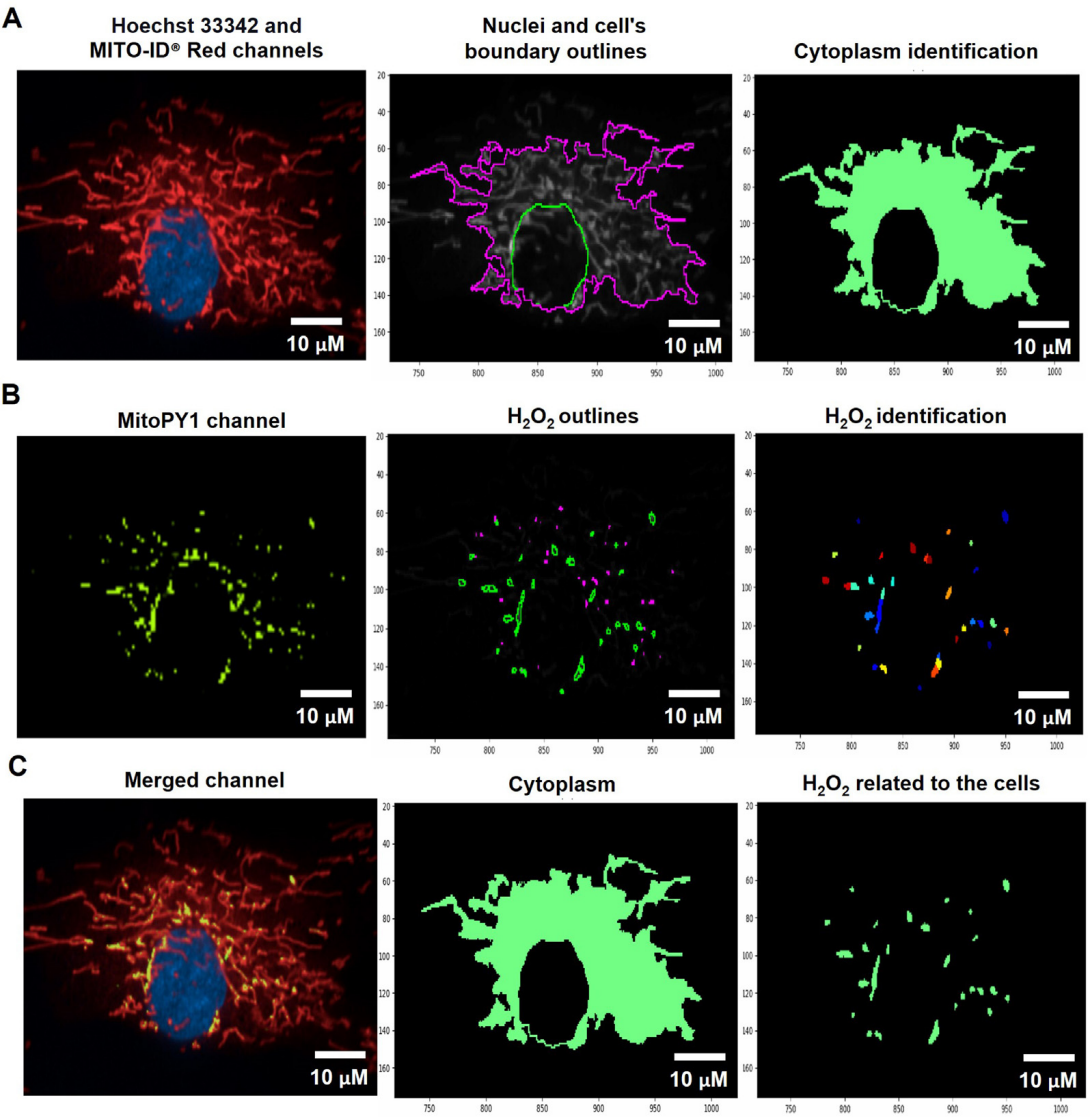
The pipeline for determining the levels of mitochondrial H_2_O_2_ by the CellProfiler program. A. The Hoechst 33342 and MITO-ID® Red channels were dedicated to identifying the Nuclei and cell's boundary outlines for the cytoplasm demarcation. B. Hydrogen peroxide (H_2_O_2_) was recalled from the green-speckled fluorescence of the MitoPY1 channels. C. Mitochondrial H_2_O_2_ was computed by relating the green-speckled fluorescence to the cells identified from Hoechst 33342 and MITO-ID® Red channels. Scale bars represent 10 µm. The pixel unit was used to scale the image.

**Fig. 3:**
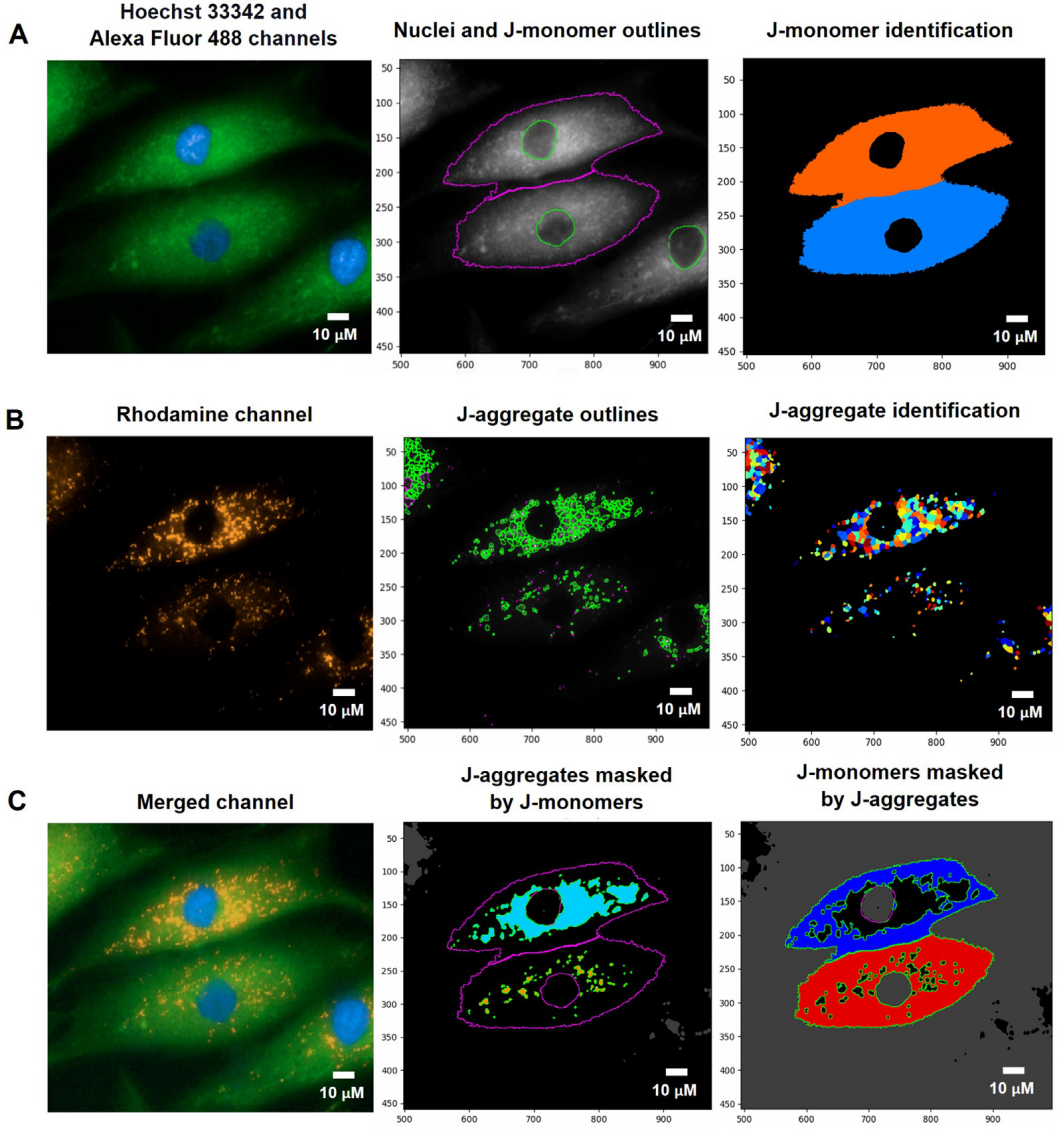
The pipeline for defining the MMP values by the CellProfiler program. A. J-monomers (green fluorescence) which produced when MMP was drop from JC-10 Alexa Fluor 488 channels associated to nuclei were specified and segmented. B. J-aggregates (orange fluorescence) indicating polarized MMP from the JC-10 Rhodamine channels were inferred. C. The areas of J-aggregates masked by J-monomers (middle image) representing orange fluorescence and the J-monomers masked by J-aggregates (right image) representing green fluorescence were identified to calculate the intensity by the Mask Object module. The MMP alterations can be evaluated quantitatively using the intensity ratio of orange-fluorescence aggregates to green-fluorescence monomers.  The pixel unit was used to scale the image.

The cytoplasmic area was defined by the calculated areas of the secondary objects (whole-cell area) minus the primary objects (nuclear area) using the Identified Tertiary Object modules, which could subtract the smaller object from the larger one (Figs. 1C, 2A, and 3A).

#### Filter objects

The Filter Object module was a method for excluding objects that had unsatisfied characteristics, for instance, clumping cells, unhealthy or degenerating cells, and other artifacts. These objects have a high intensity, a small nucleus, and an irregular shape, all of which can affect the actual results, such as representing a higher intensity. As a result, the cutoff value was adjusted to restrict the object's size, shape, eccentricity, and intensity by the Filtering measurement mode. Furthermore, we used the Image or Mask Border mode to exclude objects that are touching the image's edge because these cells do not complete the entire cell.

#### Quantification of mitochondrial H_2_O_2_

Mitochondrial hydrogen peroxide (H_2_O_2_), one of the ROS, is the parameter used to evaluate mitochondrial functions. ROS are generated when an oxygen-receiving electron escapes and becomes a superoxide anion (•O_2_^−^), an initial form of ROS, during the OXPHOS process. The •O_2_^−^ can be converted to H_2_O_2_, and H_2_O_2_ can be formed into a hydroxyl radical (•OH), which is very reactive and can cause substantial damage.

Although H_2_O_2_ produced by mitochondria plays an important role in human health and disease, it is difficult to monitor selectively inside living cells. Mitochondrial peroxy yellow 1 (MitoPY1) is a boronate-based compound designed to selectively and efficiently probe H_2_O_2_ coupled with a phosphonium moiety to target mitochondria of living cells [12]. MitoPY1 structure can alter to become a bright fluorescence when exposed to H_2_O_2_ [8, 13]. It is more sensitive to H_2_O_2_ than the commonly used MitoSOX Red. MitoSOX Red is a dihydroethidium derivative with a net positive charge that is used to detect mitochondrial •O_2_^−^. The high concentration of MitoSOX Red being used could alter mitochondrial morphology and enables it to pass through the nucleus rather than mitochondria [14]. MitoPY1 has been used to detect H_2_O_2_ in cell culture and tissues in a few recent articles [15], [16], [17]. The methods for measuring H_2_O_2_ in fibroblast culture using the MitoPY1 are described here.

To evaluate mitochondrial H_2_O_2_, the Hoechst 33342 and MITO-ID® Red channels were designated into the pipeline of organelle segmentation to reflect the nuclei and cell boundaries (Fig. 2A). The green-fluorescent intensity from the MitoPY1 channel was reinforced by using the Enhance or Suppress Feature modules; Speckle Feature Enhancement method, which heightens a region of greater intensity in comparison to its surroundings (supplementary data; Fig. S4). The green-speckled fluorescence was further applied to identify the H_2_O_2_, with the object diameter adjusted between 3 to 35 pixel units using the Identified Primary Object modules: the Adaptive, Otsu, and Three-classes thresholding strategies (Fig. 2B). This pixel's size matched the raw images of the MitoPY1 channel's heightened green-speckled fluorescence in the mitochondria, which was the reason for this. To evaluate the H_2_O_2_ levels within mitochondria, the green-speckled fluorescence (H_2_O_2_) from MitoPY1 was allocated to relate with the cells identified from the Hoechst 33342 and MITO-ID® Red channels by using the Relate Object module. The fraction of colocalized area of fluorescent signals from MitoPY1 and MITO-ID® Red was interpreted as the levels of mitochondrial H_2_O_2_ (Fig. 2C). Table 4 and supplementary data contain a table and schematics that simplify and highlight each phase of the pipeline. (Fig. S1).

**Table 4:** The process for determining mitochondrial H_2_O_2_ levels by CellProfiler program.

The pipeline of mitochondrial H_2_O_2_ quantification	
	Modules
1	Images and Metadata Extraction
2	Correction Illumination Calculate and Apply
3	Identify Primary Object: Nuclei identification (Hoechst 33342 channel)
4	Identify Secondary Object: Cell boundary identification (MITO-ID® Red channel)
5	Measure Object Size and Shape and Filter Object
6	Identify Tertiary Object: Cytoplasm identification
7	Enhance or Suppress Feature: Enhancing fluorescent intensity (MitoPY1 channel)
8	Identify Primary Object: H_2_O_2_ identification from the enhance of green-speckled fluorescence
9	Relate Objects: Relating H_2_O_2_ to the cells
10	Export to Spread Sheet: Exporting as an Excel file

#### Quantification of mitochondrial membrane potential

During electron transferring activity in mitochondria, proton pumps provide a proton gradient across the inner membrane [18]. With a few exceptions [19], the majority of the energy of the proton gradient must be described as membrane potential. Therefore, the mitochondrial membrane potential (MMP) is a key parameter for monitoring mitochondrial functional status.

We selected JC-10, a JC-1 derivative with enhanced solubility to detect the MMP. It is a cationic, lipophilic dye that is concentrated and exists in two forms: J-monomeric and J-aggregated, depending on the potential of the mitochondrial membrane. In healthy mitochondria, the protons are efficiently pumped into intermembrane space resulting in JC-10 favorably diffusing into the mitochondria due to the negative potential of inner membranes and matrix and accumulating in the reversible form of orange-fluorescent signal (J-aggregates), which indicate polarized MMP [20]. Whereas the unhealthy mitochondria (a cell is injured) with depolarized membrane potential, JC-10 exists in the cytosol and shifts to a green-fluorescent monomer [21, 22]. Therefore, changes in MMP can be detected quantitatively using the fluorescence intensity ratio of orange-fluorescent aggregates to green-fluorescent monomers.

To assess MMP status, J-monomers (green fluorescence) in the cytoplasm from JC-10 Alexa Fluor 488 channels were related to the nuclei and segmented by using the Identify Primary, Secondary, and Tertiary Object modules (Fig. 3A). To sort out the polarized mitochondria, we specified the J-aggregate by determining the threshold of orange fluorescence intensity in the JC-10 Rhodamine channel with the adjustment of object diameter between 3 to 15 pixel units using the Identified Primary Object modules: the Adaptive and Otsu methods and classifying by the Three-classes thresholding strategy (Fig. 3B). The sizes of these pixels matched the J-aggregate in the mitochondria in the raw images. The J-aggregate looked to be a little point of 3 to 15 pixels in size. The J-aggregate appeared to be a small point in the range of 3 to 15 pixels in size; otherwise, the J-aggregate might not have been detected. Subsequently, we applied the Mask object module: Keep overlapping region and Retain strategies to improve the specificity of the J-aggregate and J-monomer regions. The intensity of the J-aggregate (orange-fluorescent region masked by green region, Fig. 3C middle) and J-monomer (green-fluorescent region masked by orange region, Fig. 3C right) was further gauged by the Measure Object Intensity module. Lastly, the Calculate Math module was applied to quantify the fluorescence intensity ratio of J-aggregate to J-monomer. The table and schematic to simplify and showcase each step of the pipeline was shown in the Table 5 and supplementary data (Fig. S2).

**Table 5:** The process for determining MMP levels by the CellProfiler program.

The pipeline of MMP quantification	
	Modules
1	Images and Metadata Extraction
2	Correction Illumination Calculate and Apply
3	Identify Primary Object: Nuclei identification (Hoechst 33342 channel)
4	Identify Secondary Object: J-monomer identification (Alexa Fluor 488 channel)
5	Measure Object Size and Shape and Filter Object: Excluding the objects with unsatisfied characteristics
6	Identify Tertiary Object: Identifying the boundary of J-monomer
7	Identify Primary Object: J-aggregates identification (Rhodamine channel)
8	Mask Objects: Identifying the J-aggregate area masked by the J-monomer area
9	Mask Objects: Identifying the J-monomer area masked by the J-aggregate area
10	Measure Object Intensity: Measuring the orange intensity in the J-aggregate area
11	Measure Object Intensity: Measuring the green intensity in the J-monomer area
12	Calculate Math: Calculating the ratio of orange intensity to green intensity
13	Export to Spread Sheet: Exporting as an Excel file

#### Quantification of mitochondrial fragmentation and length

Mitochondria are dynamic organelles with the ability to change the structure by fission or fusion to maintain their function and health [23]. In this study, we used MITO-ID® Red fluorescence labeling to observe the alteration in mitochondrial morphology in any energetic state. It is a mitochondria-specific staining dye that is harmless to live cells and produces consistent fluorescence signals. According to the manufacturer's description, it also appears to be compatible with fluorescent dye use in the measurement of H_2_O_2_.

**Fig. 4:**
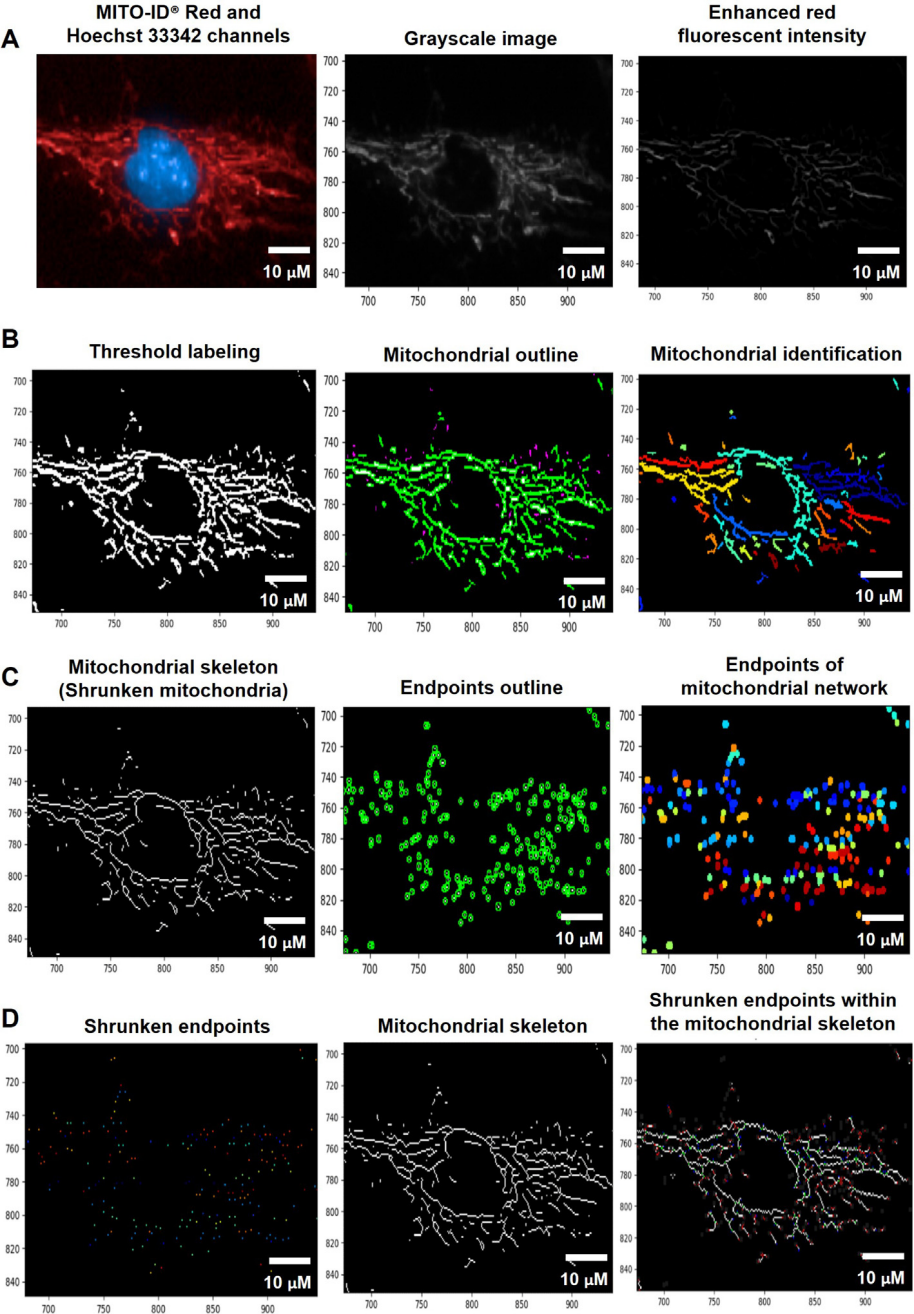
The pipeline for inferring the levels of mitochondrial fragmentation and length by the CellProfiler program. A. An image from the MITO-ID® Red channel was altered to Grayscale and enhanced the red fluorescent intensity. B. Mitochondria were identified by tagging the enhanced red fluorescence using the Thresholding strategy. C. The Morph module was utilized to shrink mitochondria into the skeleton and mark the endpoints of the skeleton. D. The mean mitochondrial length was estimated through the Measure Object Skeleton module by measuring the distance between the shrunken endpoints within the mitochondrial skeleton. Scale bars represent 10 µm. The pixel unit was used to scale the image.

To quantify mitochondrial fragmentation and length, the nuclei and cell boundaries (whole-cell area) were identified from the Hoechst 33342 and MITO-ID® Red channels using the organelle segmentation pipeline. The cytoplasmic area was further determined by subtracting the whole-cell area from the nuclear area (Fig. 1). To analyze the mitochondria, the fluorescent intensity of MITO-ID® Red channels was augmented using Enhance or Suppress Feature modules; Neurites type and Tubeness Enhancement method (Fig. 4A). The enhanced red fluorescence was further labeled using the Threshold module; Global and Minimum cross-entropy thresholding strategy to compute a single threshold value and classify pixels above the threshold as foreground (supplementary data; Fig. S5) [11]. Thereafter, the mitochondria were spotted by the Identify Primary Object module; the Global and Manual thresholding methods with a selective object diameter between 3 to 100 pixel units (Fig. 4B). The diameter of mitochondria was discovered to be around 0.2 µm [24] or 0.5 µm [25], and the length of mitochondria was determined to be around 26 µm [25] or 46 µm [26]. According to our observations, the mitochondrial diameter was less than or equivalent to 3 pixels. As a result, we assumed that 3 pixels were approximately 0.3 µm, which corresponded to the previous study's minimal mitochondrial diameter. Consequently, we set the object diameter range from 3 to 100 pixels for the identification of mitochondria.

We employed the Convert Objects to Image module with the Binary (white and black) color format to assign the mitochondria as a white color and the background as a black color. For the analysis of mitochondrial fragments, the mitochondrial granularity was accordingly assessed by the Measure Object Granularity module from white and black color format of mitochondrial images that were associated to the cytoplasm. This module could calculate the percentages of fragmented mitochondria by evaluating the divided portions of the mitochondria and designating the number of those portions as a granular spectrum with varying pixel sizes. The high levels of granularity indicated to the highly fragmented mitochondria from the fission process.

For the mitochondrial length analysis, the Morph module was conducted to examine mitochondrial morphology by shrinking the mitochondria to a single line (skeleton) (Fig. 4C left) using the Skelpe method and denoting the branch end of the mitochondrial skeleton by the Endpoint method (Fig. 4C right). The mitochondrial skeleton endpoints were then dwindled by the Expand or Shrink Object module; Shrink objects to a point (Fig. 4D left). Eventually, the Measure Object Skeleton was utilized for measuring the distance between shrunken endpoints within the mitochondrial skeleton in the pixel unit (Fig. 4D right). The table and schematic to simplify and showcase each step of the pipeline were shown in the Table 6 and supplementary data (Fig. S3).

**Table 6:** The process for determining mitochondrial fragmentation and length levels by CellProfiler program.

The pipeline of mitochondrial fragmentation and length quantification	
	Modules
1	Images and Metadata Extraction
2	Correction Illumination Calculate and Apply
3	Identify Primary Object: Nuclei identification (Hoechst 33342 channel)
4	Identify Secondary Object: Cell boundary identification (MITO-ID® Red channel)
5	Measure Object Size and Shape and Filter Object
6	Identify Tertiary Object: Cytoplasm identification
7	Enhance or Suppress Feature: Enhancing fluorescent intensity (MITO-ID® Red channel)
8	Threshold: Labeling enhanced fluorescent intensity (MITO-ID® Red channel)
9	Identify Primary Object: Mitochondrial identification from threshold labeling
10	Convert Objects to Image: Formatting the object to white and background to black
11	Measure Object Granularity: Measuring the mitochondrial granularity
12	Morph: Shrinking the mitochondria from enhanced red fluorescence to the skeleton
13	Morph: Marking the endpoints of mitochondrial skeleton
14	Identify Primary Object: Identifying the endpoints of mitochondrial skeleton
15	Expand or Shrink Object: Shrinking the endpoints of mitochondrial skeleton
16	Measure Object Skeleton: Measuring the mitochondrial length from shrunken endpoints of mitochondrial skeleton
17	Export to Spread Sheet: Exporting as an Excel file

## Statistical Analysis

We used one-way ANOVA to compare the differences of more than two groups. Next, Tukey's multiple comparisons post-hoc testing was used to analyze the differences between the mean of all possible pairs. Student's independent samples *t*-test was used to analyze the differences between the two groups by Jamovi version 1.2.22 software. Data were presented as the mean ± 95% CI (confidence interval). Statistical significances were considered when *P < 0.05, **P < 0.01, and ***P < 0.001.

### Results

#### Evaluation of mitochondrial hydrogen peroxide

**Fig. 5:**
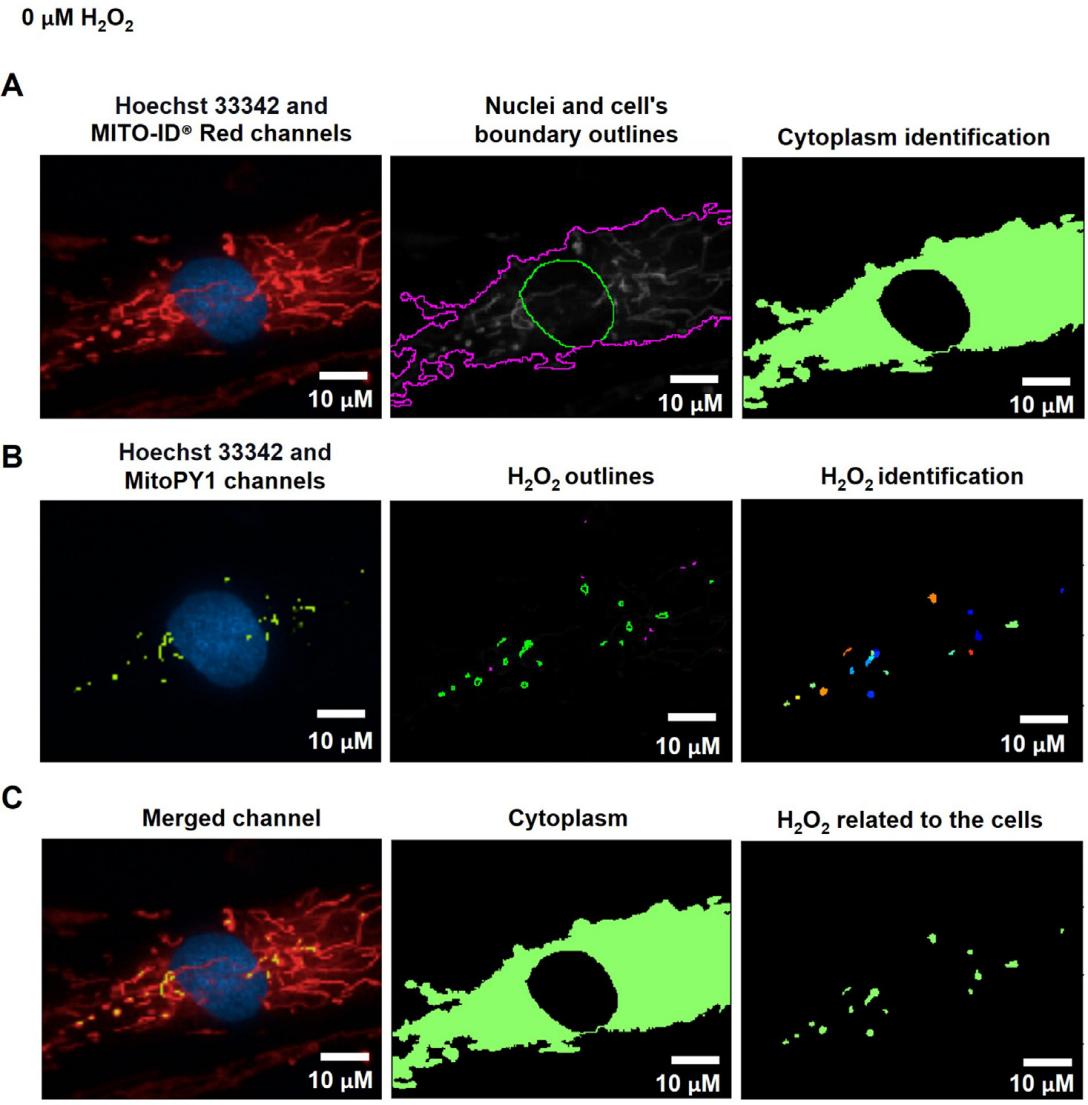
The CellProfiler program processed fluorescence pictures of untreated fibroblast cells (0 µM H_2_O_2_) through the pipeline for estimating mitochondrial H_2_O_2_ levels. The fibroblast cells were co-stained with Hoechst 33342, MITO-ID® Red, and MitoPY1 fluorescent dyes. Imaging of stained cells was visualized by the Operetta CLS with the 40x NA objective lens. A. The Hoechst 33342 (blue) and MITO-ID® Red (red) channels were dedicated to identifying the Nuclei and cell's boundary outlines for the cytoplasm identification. B. The MitoPY1 channels' green-speckled fluorescence was used to recall hydrogen peroxide (H_2_O_2_). C. Mitochondrial H_2_O_2_ was analyzed by relating the green-speckled fluorescence to the cells identified from Hoechst 33342 and MITO-ID® Red channels. Scale bars represent 10 µm.

**Fig. 6:**
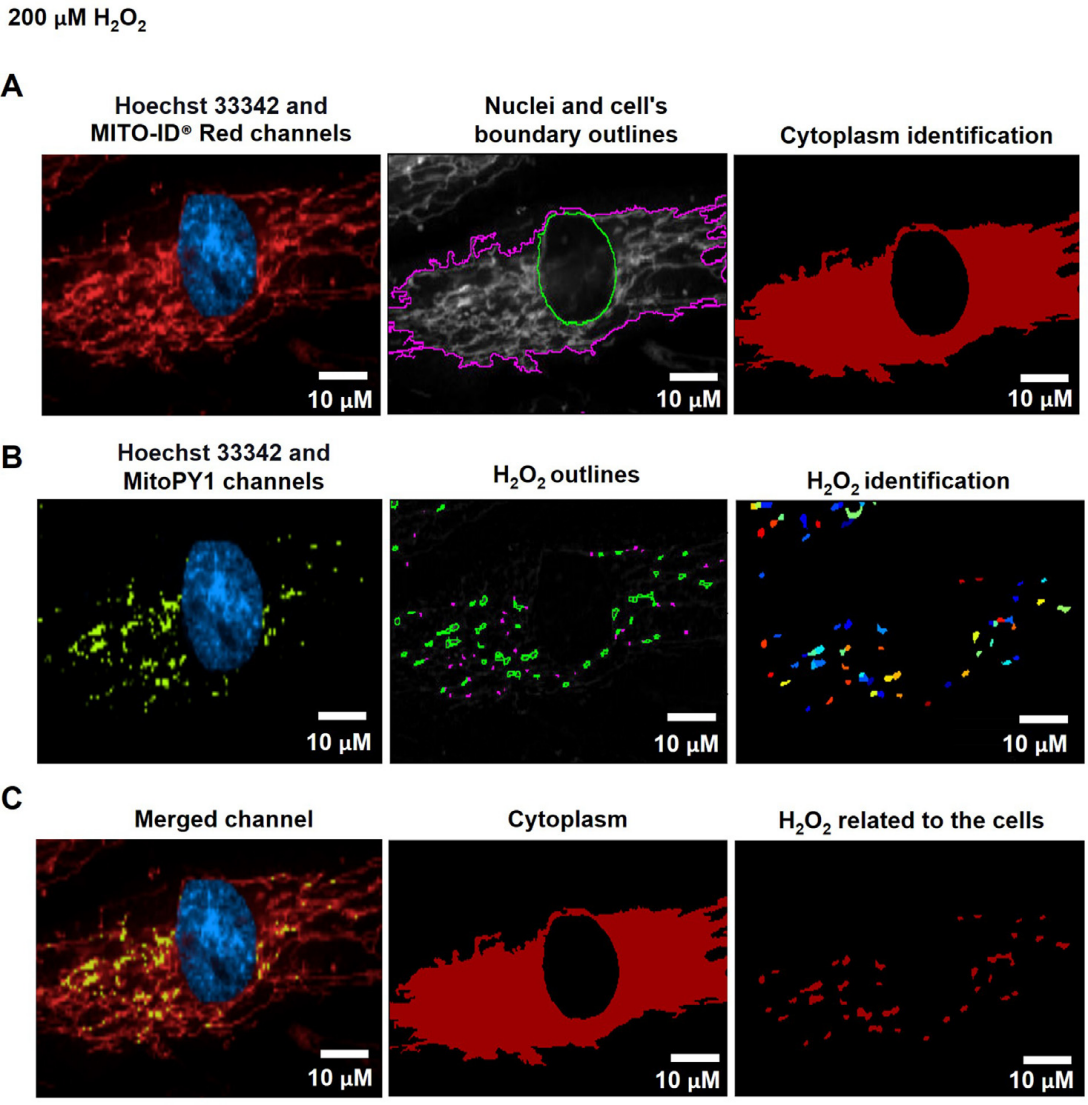
The CellProfiler program processed fluorescence pictures of fibroblast cells treated with 200 µM of H_2_O_2_ for 1 h. The fibroblast cells were co-stained with Hoechst 33342, MITO-ID® Red, and MitoPY1 fluorescent dyes. Imaging of stained cells was visualized by the Operetta CLS with the 40x NA objective lens. A. The Hoechst 33342 (blue) and MITO-ID® Red (red) channels were dedicated to identifying the nuclei and cell's boundary outlines for the cytoplasm identification. B. The MitoPY1 channels' green-speckled fluorescence was used to recall hydrogen peroxide (H_2_O_2_). C. Mitochondrial H_2_O_2_ was analyzed by relating the green-speckled fluorescence to the cells identified from Hoechst 33342 and MITO-ID® Red channels. Scale bars represent 10 µm.

**Fig. 7:**
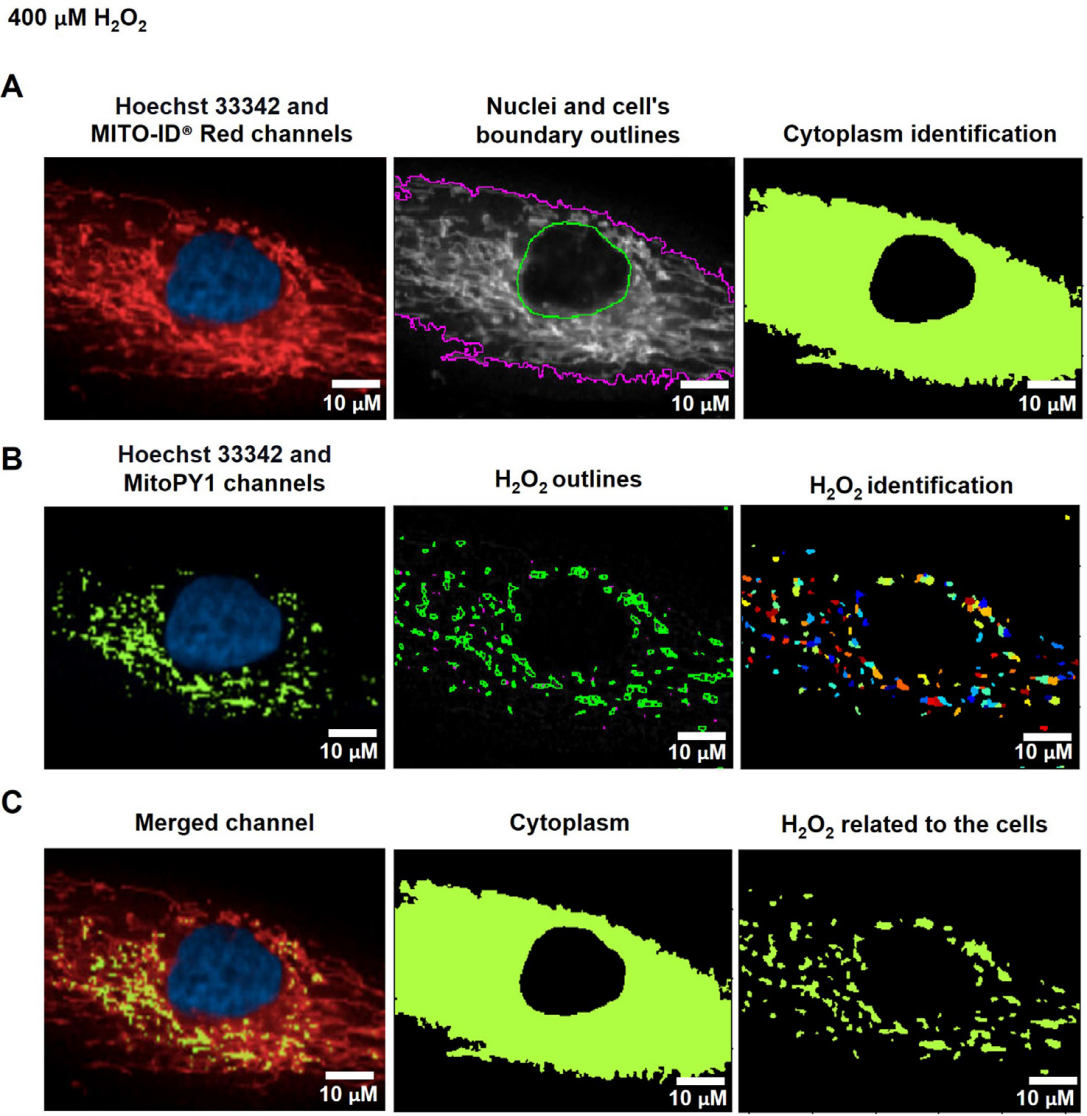
The CellProfiler program processed fluorescence pictures of fibroblast cells treated with 400 µM of H_2_O_2_ for 1 h. The fibroblast cells were co-stained with Hoechst 33342, MITO-ID® Red, and MitoPY1 fluorescent dyes. Imaging of stained cells was visualized by the Operetta CLS with the 40x NA objective lens. A. The Hoechst 33342 (blue) and MITO-ID® Red (red) channels were dedicated to identifying the Nuclei and cell's boundary outlines for the cytoplasm identification. B. The MitoPY1 channels' green-speckled fluorescence was used to recall hydrogen peroxide (H_2_O_2_). C. Mitochondrial H_2_O_2_ was analyzed by relating the green-speckled fluorescence to the cells identified from Hoechst 33342 and MITO-ID® Red channels. Scale bars represent 10 µm.

To evaluate the method of mitochondrial hydrogen peroxide (H_2_O_2_) measurement, fibroblast cells were challenged with H_2_O_2_ at 100, 200, and 400 µM in culture media for 1 h compared with the untreated cells (0 µM of H_2_O_2_). Following the treatment, the cells were co-stained with MitoPY1, MITO-ID® Red, and Hoechst 33342 dyes. The live-cell images from the Operetta CLS were processed through the pipeline for estimating mitochondrial H_2_O_2_ levels by the CellProfiler program. The fluorescence images exhibited nuclei from the Hoechst 33342 channel along with the mitochondrial network from the MITO-ID® Red channel (Figs. 5A, 6A, and 7A). The areas of green-speckled fluorescence from MitoPY1 (Figs. 6B and 7B) increased in the H_2_O_2_-treated cells compared with untreated cells (Fig. 5B). Similarly, the overlay channels revealed that H_2_O_2_ levels relating to mitochondria were elevated in the H_2_O_2_-treated cells (Figs. 6C and 7C) compared to untreated cells (Fig. 5C).

**Fig. 8:**
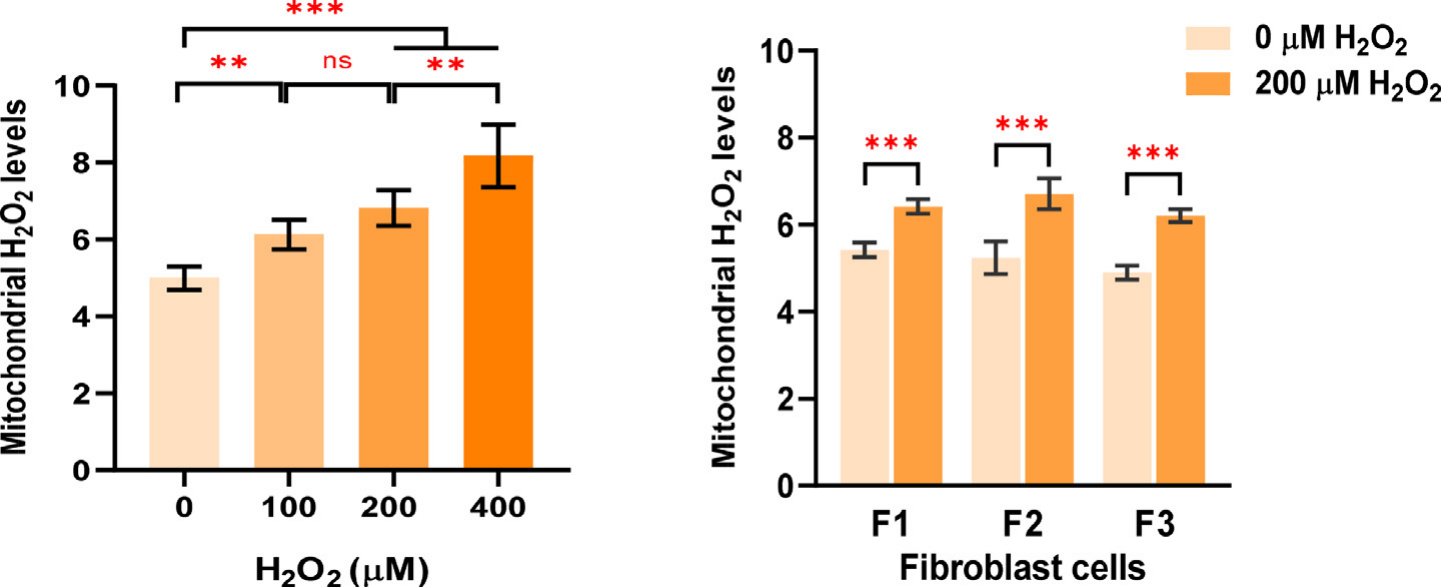
Evaluation of mitochondrial H_2_O_2_ levels. A. The mitochondrial H_2_O_2_ levels in the fibroblast cells (F2) treated with H_2_O_2_ at 100, 200, and 400 µM for 1 h compared with untreated cells (0 µM H_2_O_2_) were analyzed by the CellProfiler program. All conditions were run in triplicate wells. B. The levels of mitochondrial H_2_O_2_ in cells treated with 200 µM of H_2_O_2_ for 1 h compared with untreated cells were assessed in individual fibroblast samples (F1, F2, and F3). All conditions were run in duplicate wells. Each treatment condition was represented by 48 - 60 fields per well (15 - 40 cells per field), leading to 2,160 – 7,200 cells (for triplicate wells) and 1,440 – 4,800 cells (for duplicate wells) overall for each condition. Each plate was treated as an individual experiment, n=1 per condition. The graphs were presented as mean values with 95% CI. Statistical significance is analyzed using ANOVA with Tukey's multiple comparison post-test and the student's independent sample *t*-test. Statistical significance was considered when *P < 0.05, **P < 0.01 and ***P < 0.001.

The colocalized areas disclosed the increasing levels of mitochondrial H_2_O_2_ in a dose-dependent manner of H_2_O_2_ treatment. The mitochondrial H_2_O_2_ levels significantly increased in the cells treated with H_2_O_2_ at 100, 200, and 400 µM compared with untreated cells (Fig. 8A). Moreover, in distinct fibroblast samples (F1, F2, and F3), the mitochondrial H_2_O_2_ levels were considerably elevated in cells treated with H_2_O_2_ at 200 µM compared to untreated cells (Fig. 8B). However, the number of green-speckled fluorescent areas in images (Fig. 5, 6, and 7) appeared to be greater than the quantitative results of mitochondrial H_2_O_2_ levels (Fig. 8). The reason for this was that the Relate Object module might select just the high intensity of green-speckled fluorescence for relating to the nucleus, where mitochondrial H_2_O_2_ largely accumulated around the nucleus (perinuclear) while peripheral areas were mostly low-intensity.

**Fig. 9:**
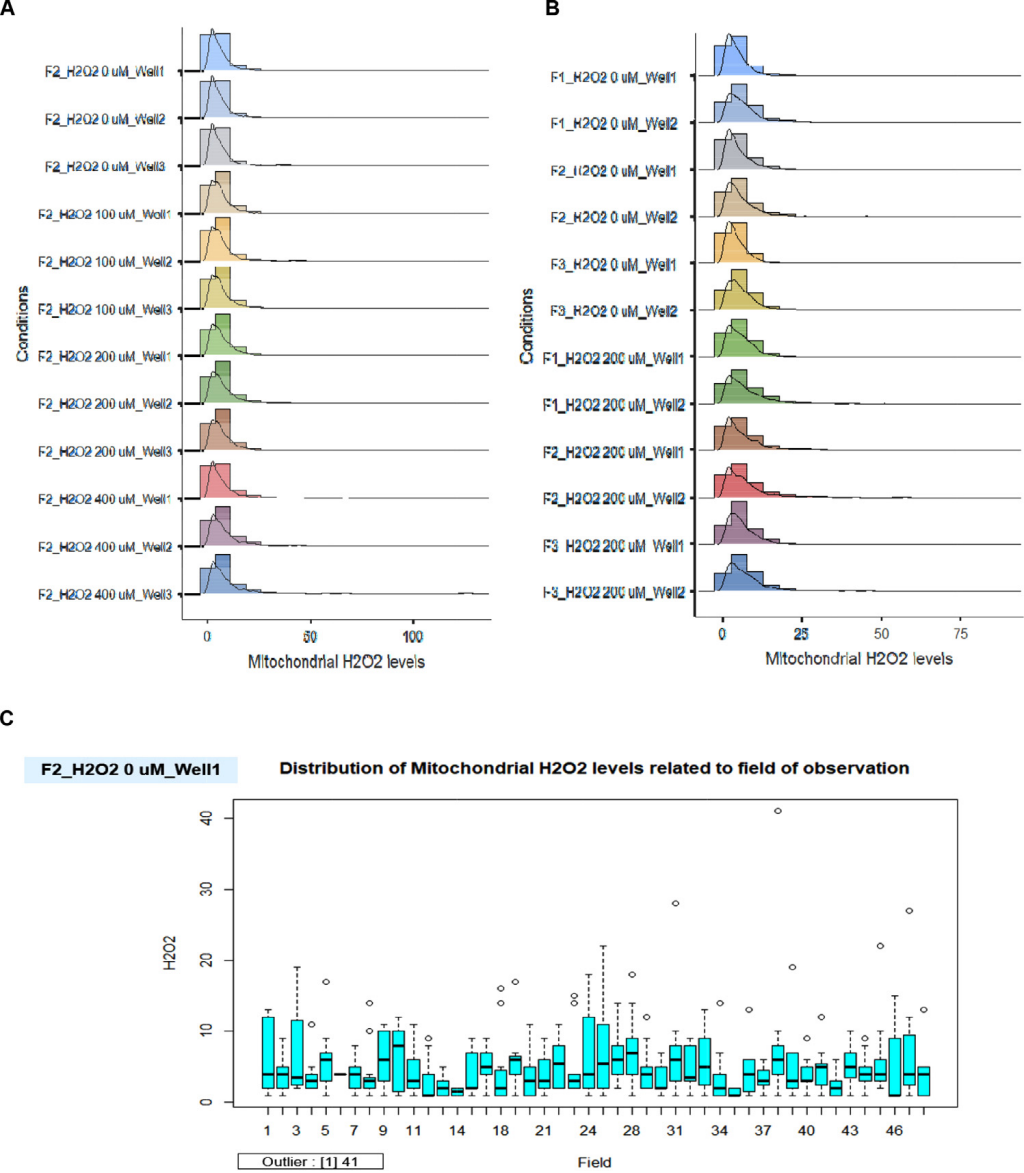

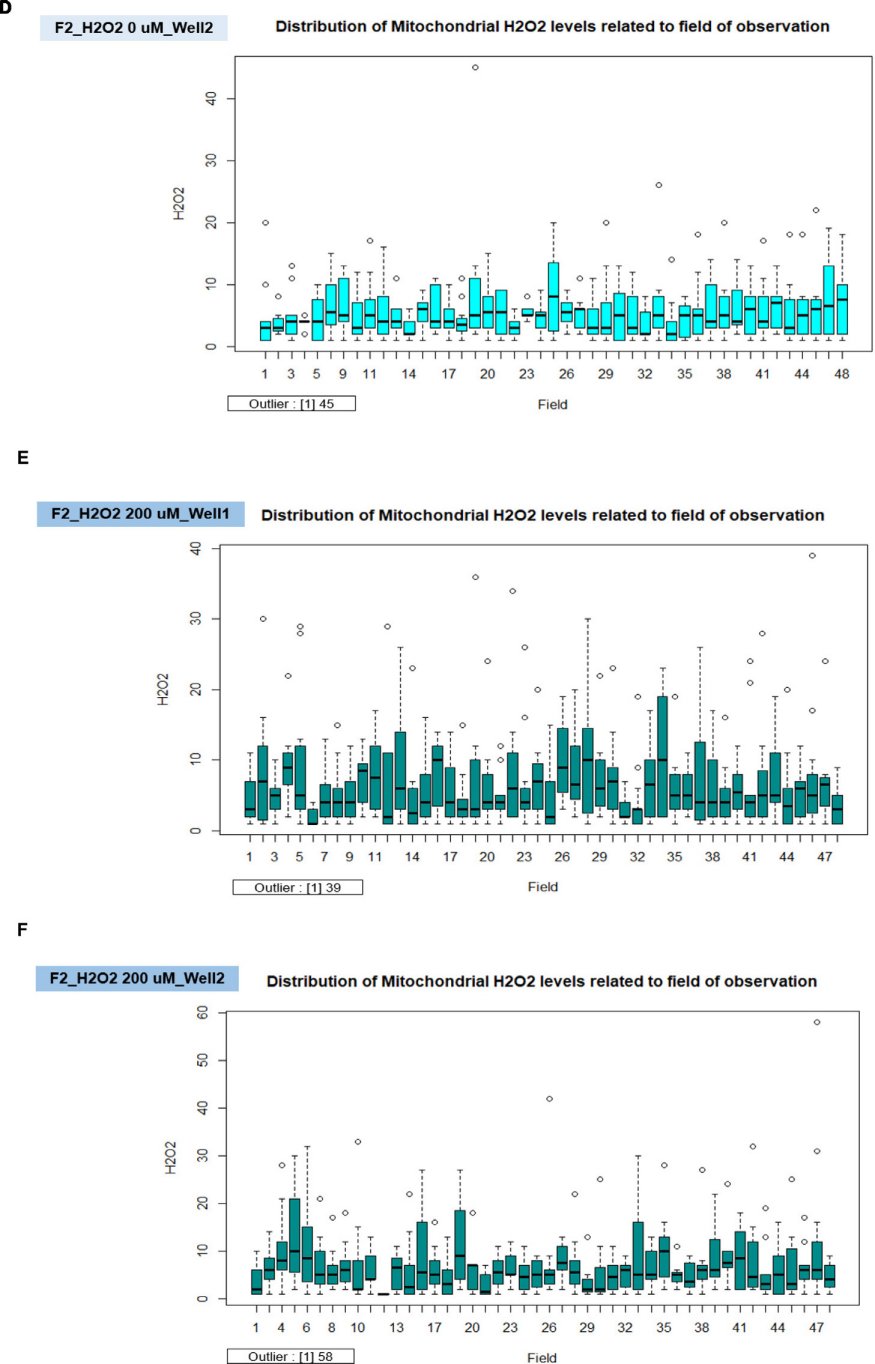
The validation of robust quality control in each well and field for the measurement of mitochondrial H_2_O_2_ levels. A. The distribution of mitochondrial H_2_O_2_ levels for the titration of H_2_O_2_ concentrations related to well. B. The distribution of mitochondrial H_2_O_2_ levels related to well for the comparison of 200 µM of H_2_O_2_ treated cells and untreated cells in individual fibroblast samples. C. The distribution of mitochondrial H_2_O_2_ levels related to the field of observation in the conditions of untreated cells (0 µM of H_2_O_2_) in well 1 of F2 fibroblast sample. D. The distribution of mitochondrial H_2_O_2_ levels related to the field of observation in the conditions of untreated cells (0 µM of H_2_O_2_) in well 2 of F2 fibroblast sample. E. The distribution of mitochondrial H_2_O_2_ levels related to the field of observation in the conditions of 200 µM of H_2_O_2_ treated cells in well 1 of F2 fibroblast sample. F. The distribution of mitochondrial H_2_O_2_ levels related to the field of observation in the conditions of 200 µM of H_2_O_2_ treated cells in well 2 of F2 fibroblast sample.

To assess the quality control, we displayed the distribution of mitochondrial H_2_O_2_ levels in the triplicate wells for the titration of H_2_O_2_ concentrations (Fig. 9A) and the duplicate wells for the comparison of 200 µM of H_2_O_2_ treated cells and untreated cells in individual fibroblast samples (Fig. 9B). In all conditions, the well-related distribution graph of mitochondrial H_2_O_2_ levels displayed a left-skewed distribution. There was a minor difference between the wells (1, 2, and 3) (Fig. 9A and B). For the distribution of H_2_O_2_ levels related to the field of observation, for example, in the conditions of F2_H_2_O_2_ 0 µM_Well 1 (Fig. 9C), F2_H_2_O_2_ 0 µM_Well 2 (Fig. 9D), F2_H_2_O_2_ 200 µM_Well 1 (Fig. 9E), and F2_H_2_O_2_ 200 µM_Well 2 (Fig. 9F), the data revealed a minor difference between the fields. We next utilized the R tool to find the outlier of the data points in each well, and we noticed that each condition in Fig. 9C - F had only one outlier.

#### Evaluation of mitochondrial membrane potential levels

**Fig. 10:**
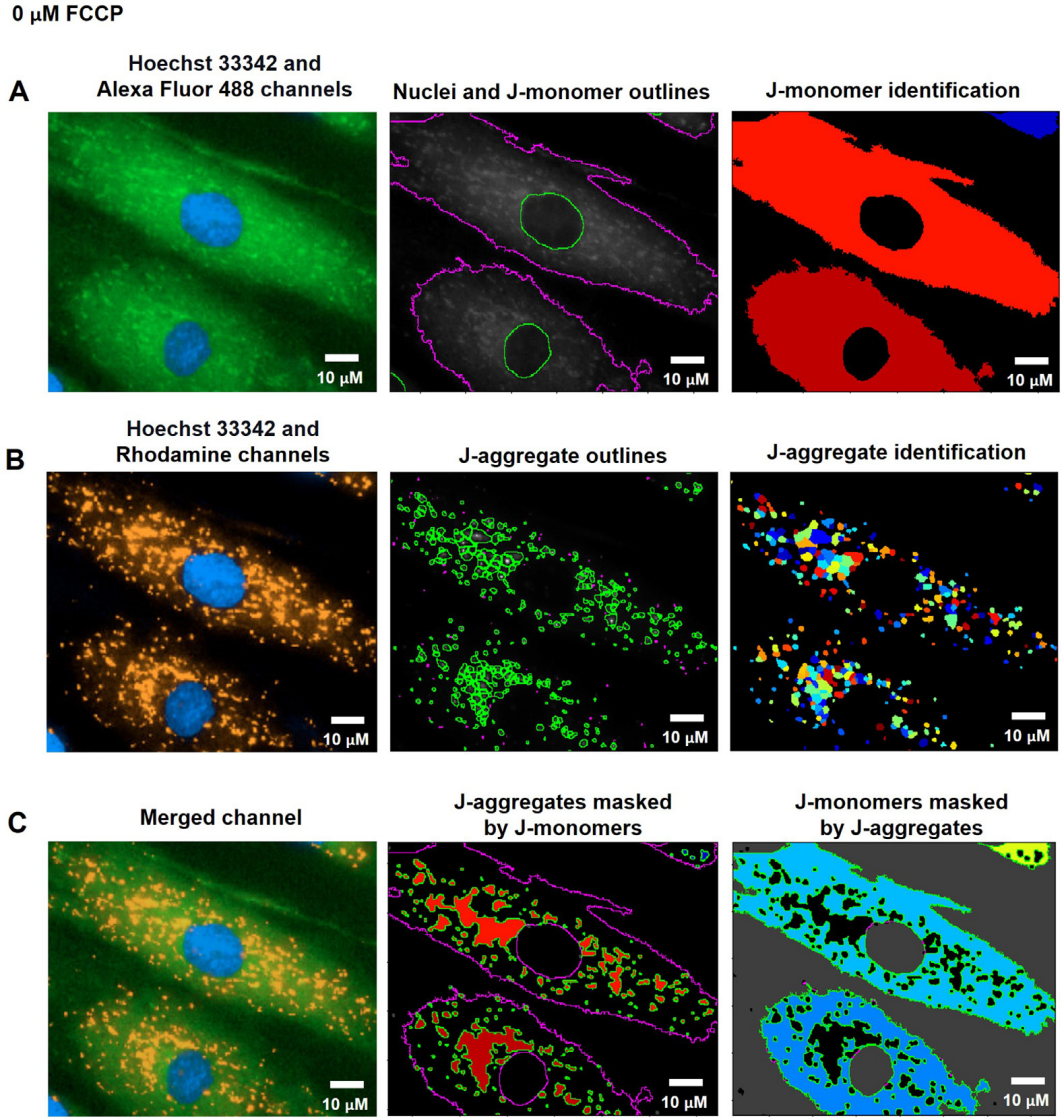
CellProfiler program processed fluorescence pictures of untreated fibroblast cells (ethanol, 0 µM of FCCP) via the pipeline for measuring MMP levels. The fibroblast cells were co-stained with Hoechst 33342 and JC-10 fluorescent dyes. Imaging of stained cells was visualized by the Operetta CLS with the 40x NA objective lens. A. J-monomers (green-fluorescence) from JC-10 Alexa Fluor 488 channels associated with nuclei were specified and segmented. B. J-aggregates (orange-fluorescence) indicating polarized MMP from the JC-10 Rhodamine channels were inferred. C. The areas of J-aggregates masked by J-monomers (middle image) representing orange-fluorescence and the J-monomers masked by J-aggregates (right image) representing green-fluorescence were identified to calculate the intensity by the Mask Object module. The MMP alterations can be evaluated quantitatively using the intensity ratio of orange-fluorescence aggregates to green-fluorescence monomers. Scale bars represent 10 µm.

**Fig. 11:**
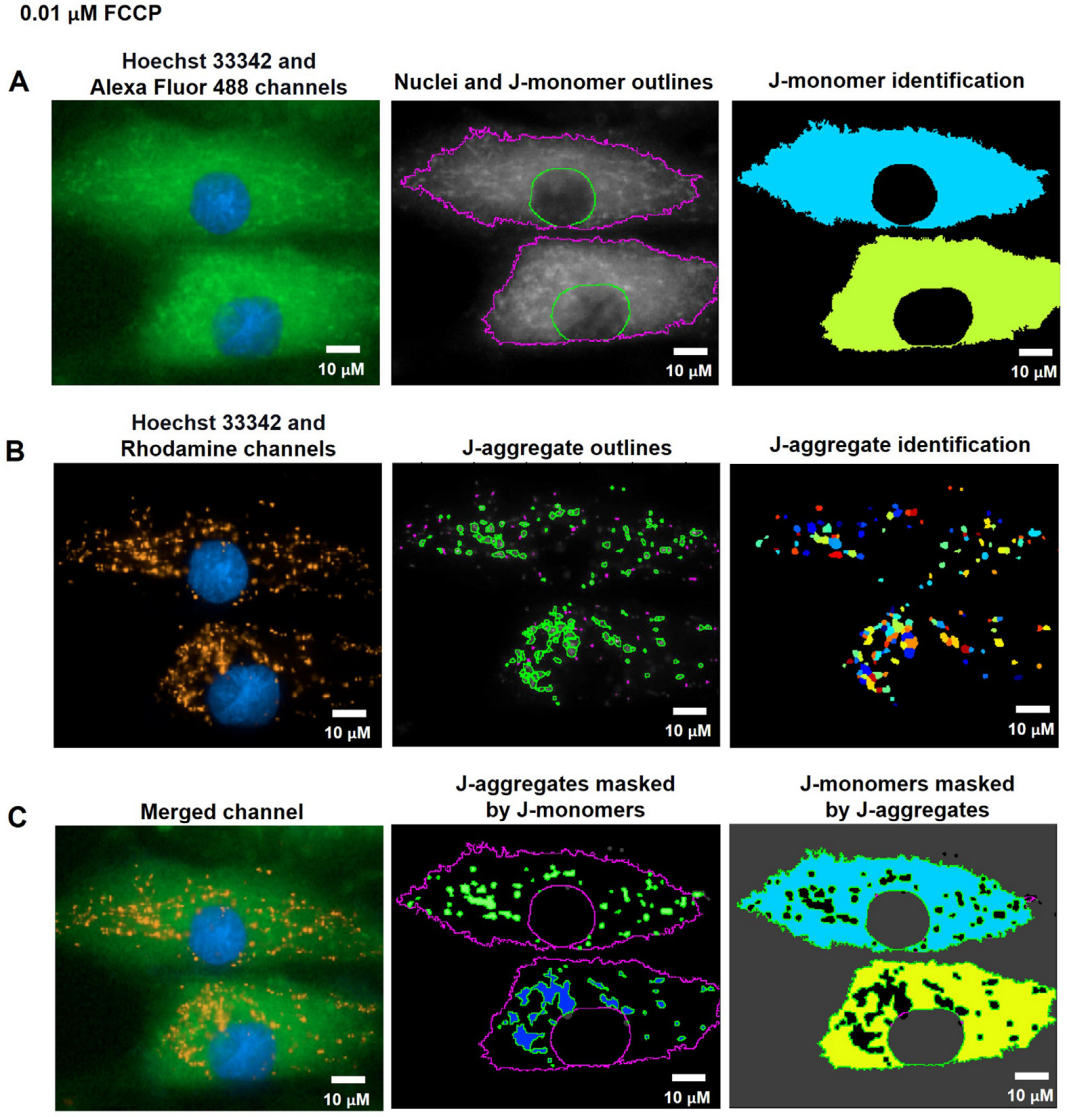
CellProfiler program processed fluorescence pictures of fibroblast cells treated with 0.01 µM of FCCP for 1 h via the pipeline for measuring MMP levels. The fibroblast cells were co-stained with Hoechst 33342 and JC-10 fluorescent dyes. Imaging of stained cells was visualized by the Operetta CLS with the 40x NA objective lens. A. J-monomers (green-fluorescence) from JC-10 Alexa Fluor 488 channels associated with nuclei were specified and segmented. B. J-aggregates (orange-fluorescence) indicating polarized MMP from the JC-10 Rhodamine channels were inferred. C. The areas of J-aggregates masked by J-monomers (middle image) representing orange-fluorescence and the J-monomers masked by J-aggregates (right image) representing green-fluorescence were identified to calculate the intensity by the Mask Object module. The MMP alterations can be evaluated quantitatively using the intensity ratio of orange-fluorescence aggregates to green-fluorescence monomers. Scale bars represent 10 µm.

**Fig. 12:**
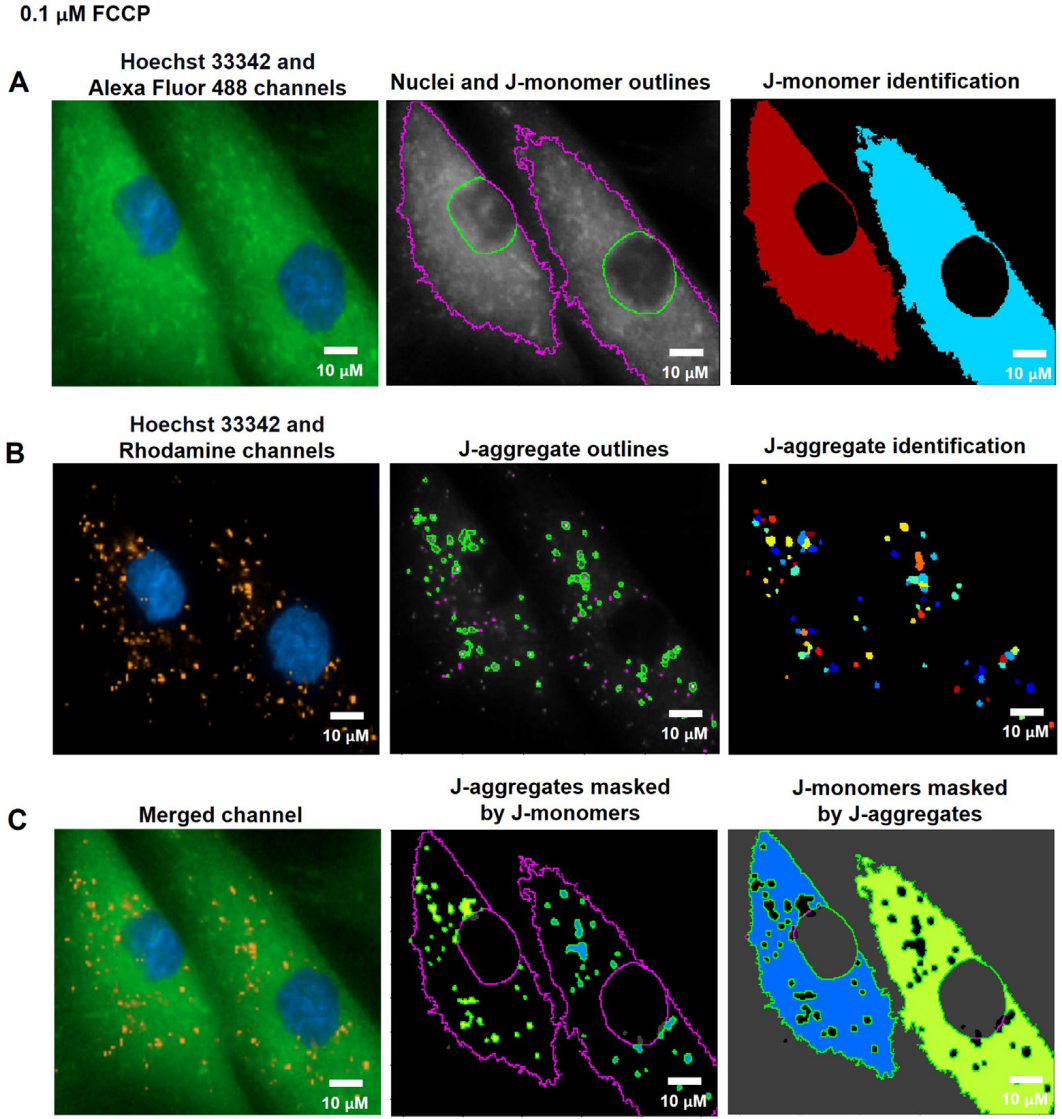
CellProfiler program processed fluorescence pictures of fibroblast cells treated with 0.1 µM of FCCP for 1 h via the pipeline for measuring MMP levels. The fibroblast cells were co-stained with Hoechst 33342 and JC-10 fluorescent dyes. Imaging of stained cells was visualized by the Operetta CLS with the 40x NA objective lens. A. J-monomers (green-fluorescence) from JC-10 Alexa Fluor 488 channels associated with nuclei were specified and segmented. B. J-aggregates (orange-fluorescence) indicating polarized MMP from the JC-10 Rhodamine channels were inferred. C. The areas of J-aggregates masked by J-monomers (middle image) representing orange-fluorescence and the J-monomers masked by J-aggregates (right image) representing green-fluorescence were identified to calculate the intensity by the Mask Object module. The MMP alterations can be evaluated quantitatively using the intensity ratio of orange-fluorescence aggregates to green-fluorescence monomers. Scale bars represent 10 µm.

**Fig. 13:**
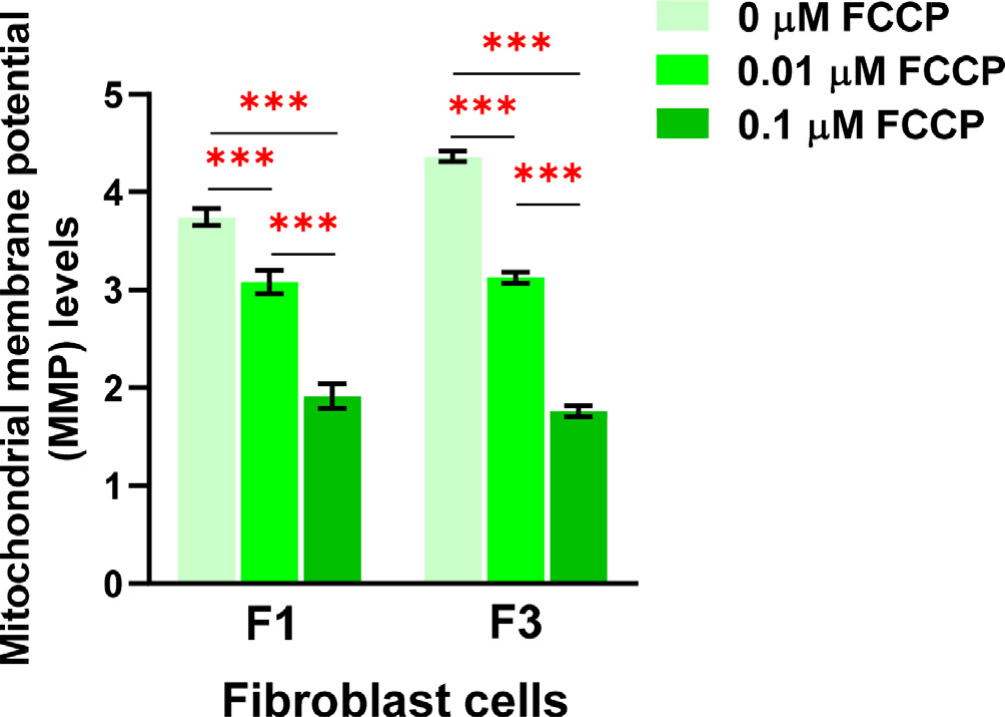
Evaluation of mitochondrial membrane potential (MMP) levels. The MMP levels was calculated from the ratios of orange-fluorescent aggregates to green-fluorescent monomers in individual fibroblast samples (F1 and F3) treated with 0.01 and 0.1 µM of FCCP compared with vehicle control (ethanol, 0 µM of FCCP) for 1 h. All conditions were run in duplicate wells. Each treatment condition was represented by 60 - 80 fields per well (15 - 40 cells per field), leading to 3,600 – 12,800 cells (for four wells) and 1,800 – 6,400 cells (for duplicate wells) overall for each condition. Each plate was treated as an individual experiment, n=1 per condition. The graphs were presented as mean values with 95% CI. Statistical significance is analyzed using ANOVA with Tukey's multiple comparison post-test. Statistical significance was considered when *P < 0.05, **P < 0.01 and ***P < 0.001.

FCCP (Carbonyl cyanide-*p*-trifluoromethoxyphenylhydrazone) is widely used in mitochondrial research as an uncoupling reagent. FCCP disrupts the proton gradient between the matrix and intermembrane space of mitochondria, resulting in a decrease in MMP [27]. In this study, we treated the cells with 0.01, 0.1 µM of FCCP, or ethanol (vehicle control) in the culture media for 1 h. The cells were then stained with Hoechst 33342 and JC-10 dyes after treatment. The CellProfiler program was used to run live-cell images from the Operetta CLS through a pipeline for determining MMP levels. The nuclei from the Hoechst 33342 channel coexisted with the green-fluorescence (J-monomers) from the JC-10 Alexa Fluor 488 channel in the fluorescence images (Figs. 10A, 11A, and 12A). The fluorescent images showed that vehicle control cells (0 µM of FCCP) (Figs. 10B) exhibited more orange-fluorescence (J-aggregates) representing polarized MMP than the cells treated with FCCP at 0.01 and 0.1 µM, as observed from the JC-10 Rhodamine channels (Figs. 11B and 12B). As well, the merged channels revealed that areas of J-aggregates masked by J-monomers (Fig. 10C middle image) increased more than in the FCCP-treated cells (Figs. 11C and 12C middle image). The intensity ratios of orange-fluorescent aggregates to green-fluorescent monomers, which indicated the MMP levels, significantly decreased in cells treated with FCCP with a dose-dependence compared to vehicle control cells in distinct fibroblast samples (F1 and F3) (Fig. 13). Our findings indicated a more sensitive detection of MMP changes in response to 0.01 and 0.1 µM of FCCP treatment, compared to the previous study that used TMRM fluorescence in rat ventricular myocytes [28].

**Fig. 14:**
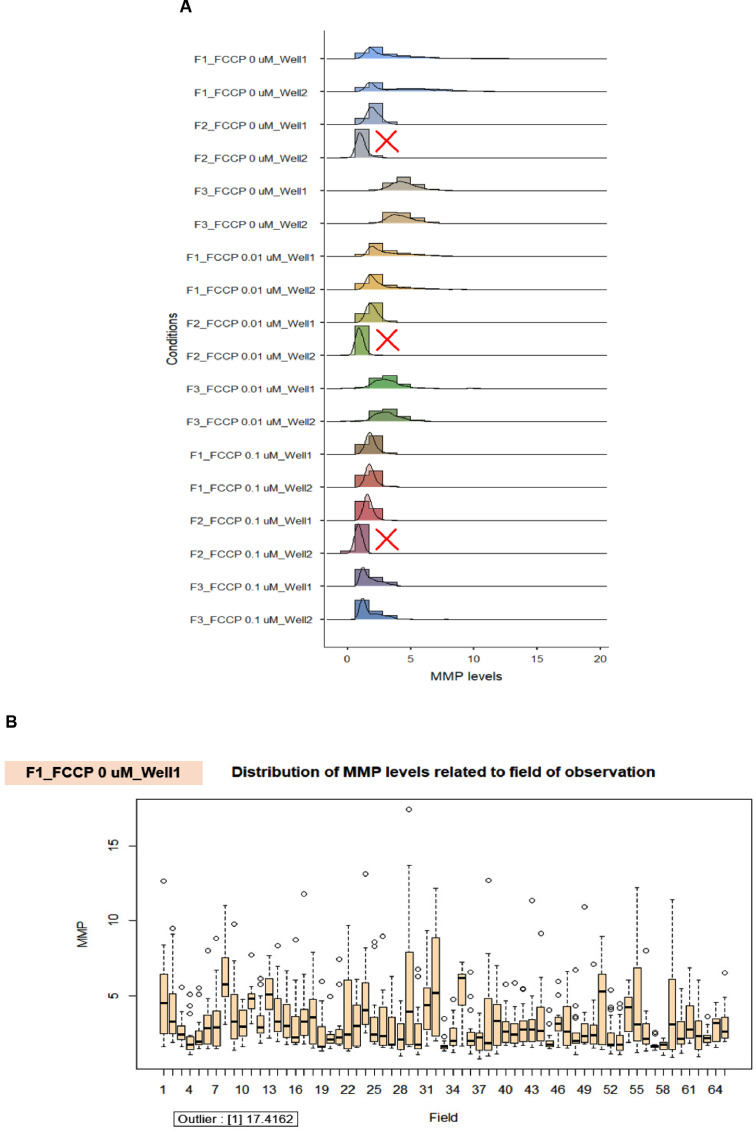

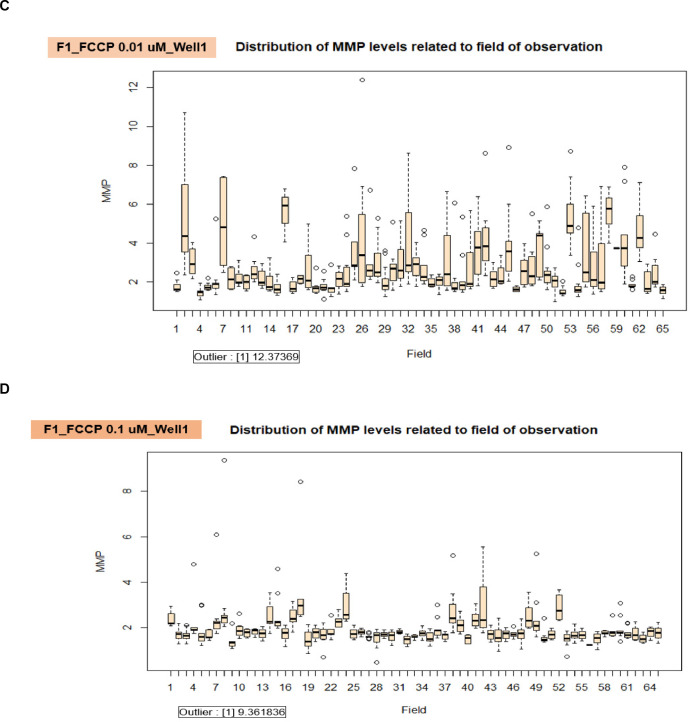
The validation of robust quality control in each well and field for the measurement of MMP levels. A. The well-related distribution of MMP levels in the duplicate wells of the 0.01 µM and 0.1 µM of FCCP treated cells and the untreated cells in individual fibroblast samples (F1, F2, and F3). Due to the obvious lower range when compared to well 1 and other samples within the same conditions, the results in well 2 of F2 samples in all conditions were excluded (red cross mark). B. The distribution of MMP levels related to the field of observation in the conditions of the vehicle control (0 µM of FCCP) in well 1 of F1 fibroblast sample. C. The distribution of MMP levels related to the field of observation in the conditions of 0.01 µM of FCCP treated cells in well 1 of F1 fibroblast sample. D. The distribution of MMP levels related to the field of observation in the conditions of 0.1 µM of FCCP treated cells in well 1 of F1 fibroblast sample.

We displayed the well-related distribution of MMP levels in the duplicate wells of the 0.01 µM and 0.1 µM of FCCP treated cells and the untreated cells in individual fibroblast samples (Fig. 14A) for validation of MMP measurement. We discovered the anomalous data in well 2 of F2 samples in the conditions of F2_FCCP 0 µM_Well 2, F2_FCCP 0.01 µM_Well 2, and F2_FCCP 0.1 µM_Well 2 (the red cross mark in Fig. 14A). When compared to well 1 and the other samples (F1 and F3) in the identical settings, the distribution of MMP levels in well 2 of the F2 samples was in the lower range. Therefore, the results from F2 samples in measuring of MMP levels were omitted. For the observation of the distribution of MMP levels associated with the field, the data showed minor variation between the fields, such as in the conditions of F1_FCCP 0 µM Well 1 (Fig. 14B), F1_FCCP 0.01 µM Well 1 (Fig. 14C), and F1_FCCP 0.1 µM Well 1 (Fig. 14D). We further utilized the R program to find the outlier in each well's data, and we noticed each condition (Fig. 14B – D) had only one outlier.

#### Evaluation of mitochondrial fragmentation and length

**Fig. 15:**
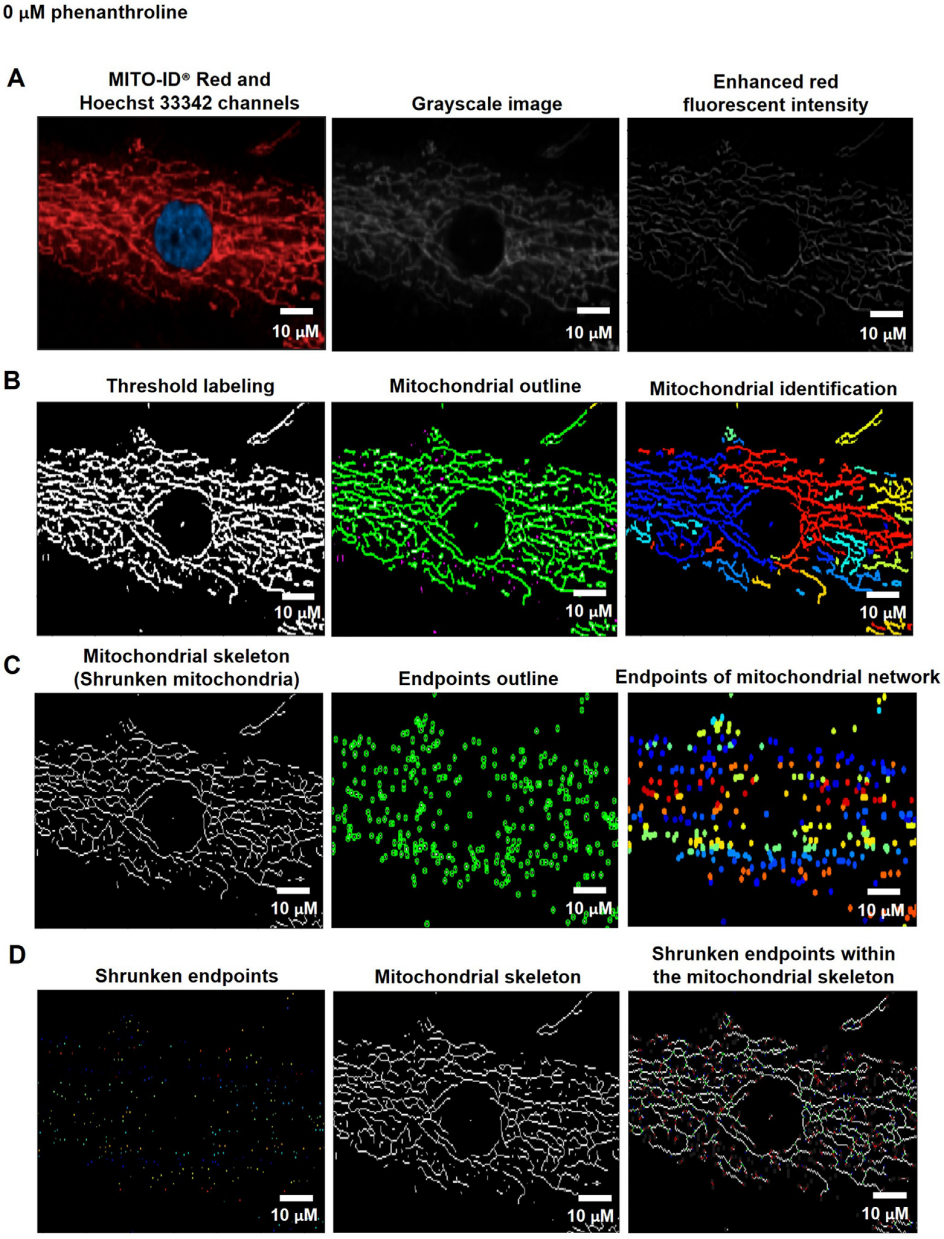
CellProfiler program processed fluorescence pictures of untreated fibroblast cells (ethanol, 0 µM of phenanthroline) through the pipeline for analyzing the levels of mitochondrial fragmentation and length. The fibroblast cells were co-stained with Hoechst 33342 and MITO-ID® Red fluorescent dyes. Imaging of stained cells was visualized by the Operetta CLS with the 40x NA objective lens. A. The image from the MITO-ID® Red channel was altered to Grayscale and enhanced the red fluorescent intensity. B. Mitochondria were identified by tagging the enhanced red fluorescence using the Thresholding strategy. C. The Morph module was utilized to shrink mitochondria into the skeleton and mark the endpoints of the skeleton. D. The mean mitochondrial length and fragmentation per cell were estimated through the Measure Object Skeleton module by measuring the distance between the shrunken endpoints within the mitochondrial skeleton. Scale bars represent 10 µm.

**Fig. 16:**
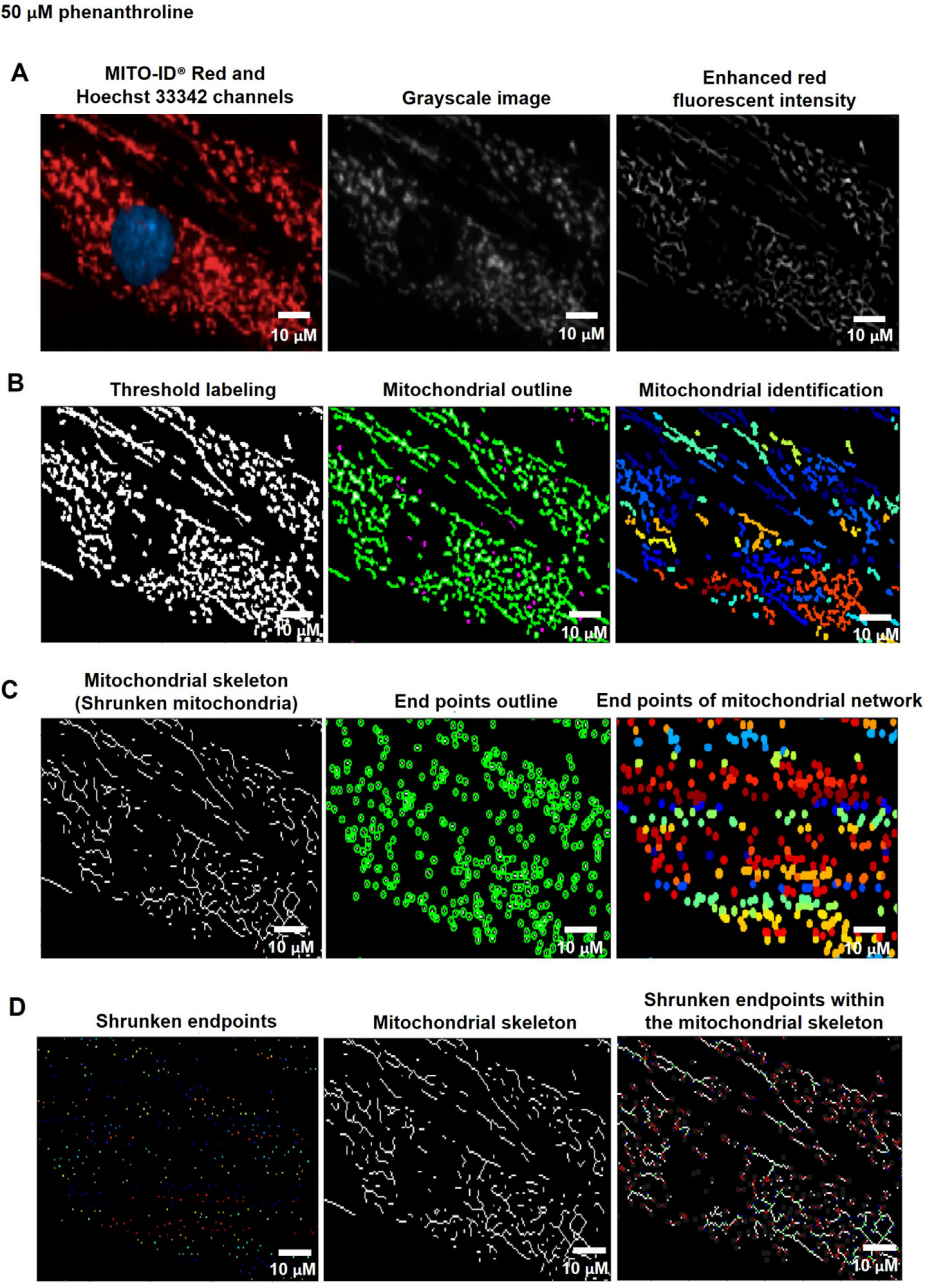
CellProfiler program processed fluorescence pictures of fibroblast cells treated with 50 µM of phenanthroline for 4 through the pipeline for analyzing the levels of mitochondrial fragmentation and length. The fibroblast cells were co-stained with Hoechst 33342 and MITO-ID® Red fluorescent dyes. Imaging of stained cells was visualized by the Operetta CLS with the 40x NA objective lens. A. The image from the MITO-ID® Red channel was altered to Grayscale and enhanced the red fluorescent intensity. B. Mitochondria were identified by tagging the enhanced red fluorescence using the Thresholding strategy. C. The Morph module was utilized to shrink mitochondria into the skeleton and mark the endpoints of the skeleton. D. The mean mitochondrial length and fragmentation per cell were estimated through the Measure Object Skeleton module by measuring the distance between the shrunken endpoints within the mitochondrial skeleton. Scale bars represent 10 µm.

To evaluate the method for assessing mitochondrial morphology, we selected 1,10-phenanthroline, which has been shown to induce mitochondrial fragmentation in HeLa cells. Reportedly, phenanthroline stimulates DRP1, a protein that operates mitochondrial fission [29]. In this study, the fibroblast cells were treated with 50 µM of phenanthroline or ethanol (vehicle control) in culture media for 4 h. After drug treatment, cells were stained with MITO-ID® Red and Hoechst-33342. The CellProfiler program was used to determine the levels of mitochondrial fragmentation and length. The fluorescent images from MITO-ID® Red channel were converted to grayscale and the red fluorescent intensity was increased (Figs. 15A and 16A). The mitochondria were then identified from the threshold labeling (Figs. 15B and 16B). The mitochondrial networks were reduced to a single line (skeleton) and then shrank into the endpoints (Figs. 15C and 16C). The distance between shrinking endpoints inside the mitochondrial skeleton was related to the cytoplasm (Figs. 15D and 16D). The length between endpoints inside the mitochondrial skeleton in vehicle control (Fig. 15) was obviously longer than in cells treated with 50 µM of phenanthroline (Fig. 16). Furthermore, the number of fragmented mitochondria in phenanthroline-treated cells (Fig. 16) seemed to higher than in vehicle control (Fig. 15).

**Fig. 17:**
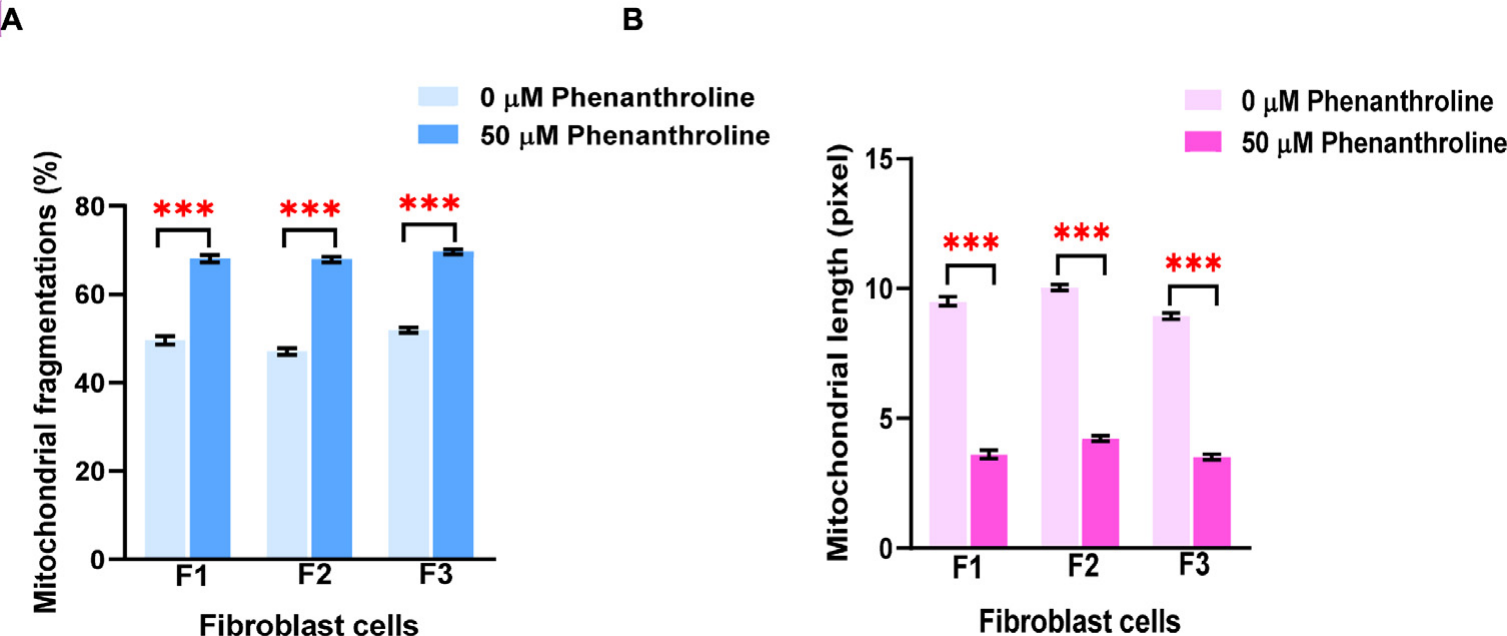
Evaluation of mitochondrial fragmentation and length levels. A. The percentage of mitochondrial fragmentation (granularity) was measured in the individual fibroblast cells (F1, F2, and F3) treated with 50 µM of phenanthroline compared with vehicle control (ethanol, 0 µM of phenanthroline) for 4 h. B. The levels of mitochondrial length (pixel) were assessed in fibroblast samples (F1, F2, and F3) treated with 50 µM of phenanthroline compared with vehicle control. All conditions were run in duplicate wells. Each treatment condition was represented by 60-80 fields per well (15-40 cells per field), leading to 1,800 – 6,400 cells (for duplicate wells) overall for each condition. Each plate was treated as an individual experiment, n=1 per condition. The graphs were presented as mean values with 95% CI. Statistical significance is analyzed using the student's independent sample *t*-test. Statistical significance was considered when *P < 0.05, **P < 0.01 and ***P < 0.001.

**Fig. 18:**
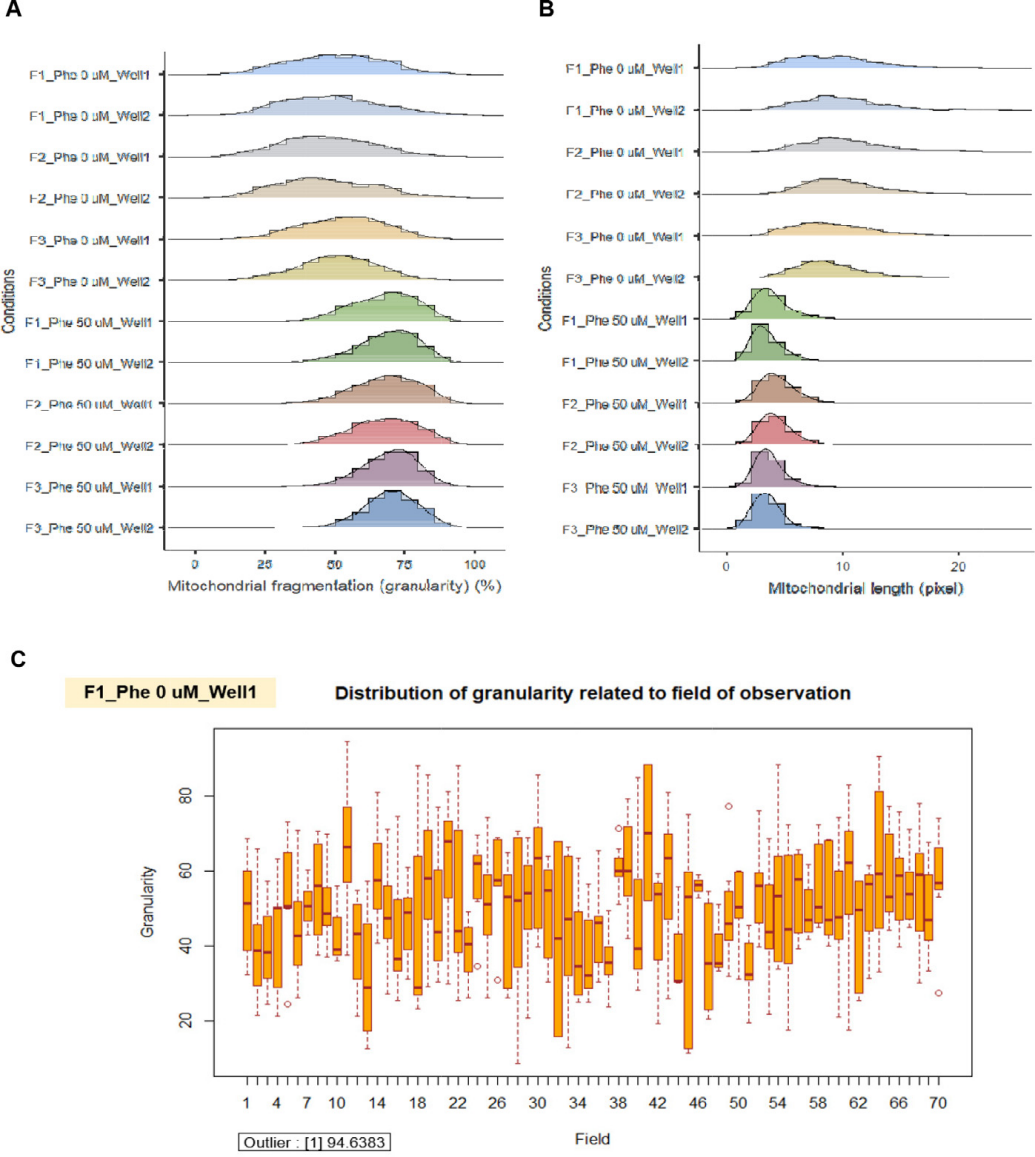

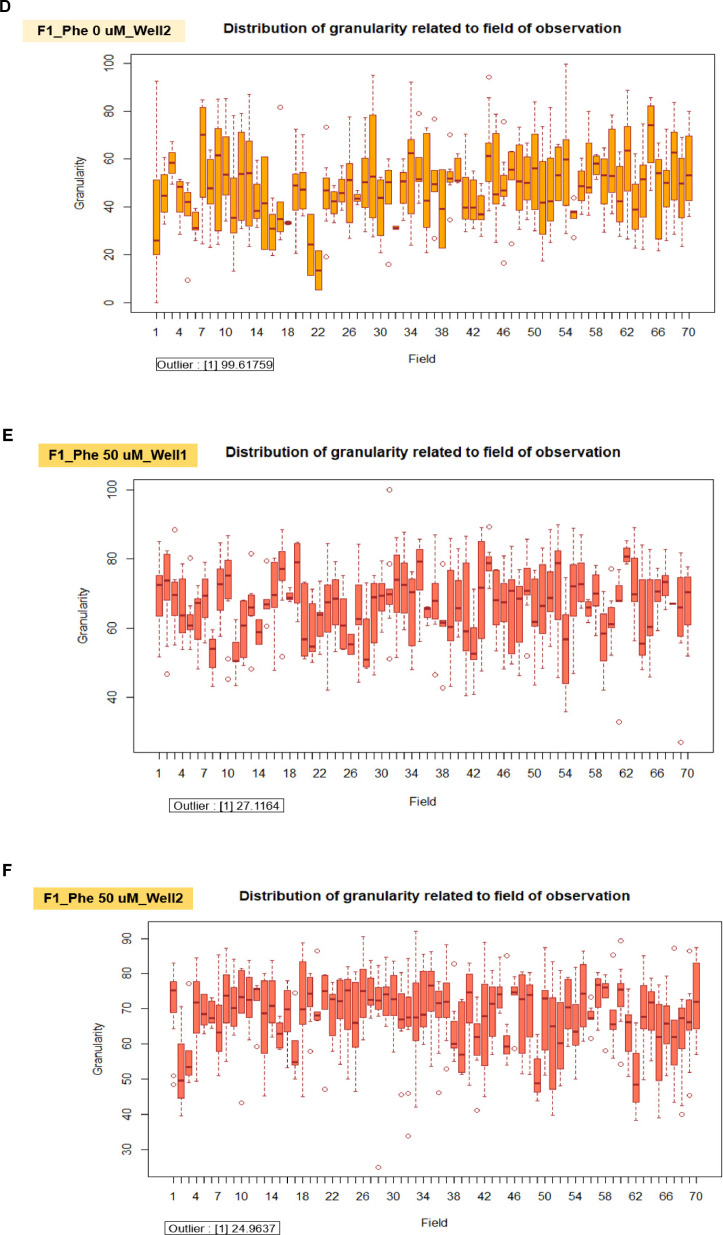

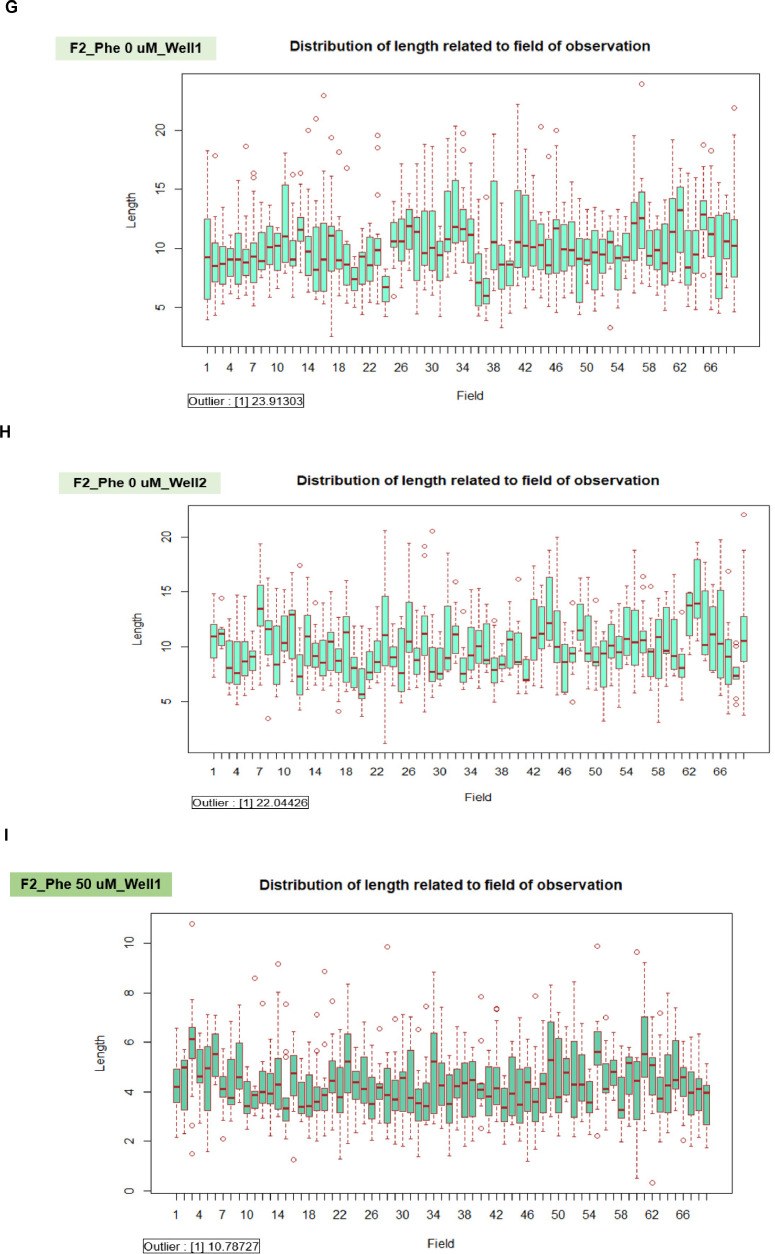

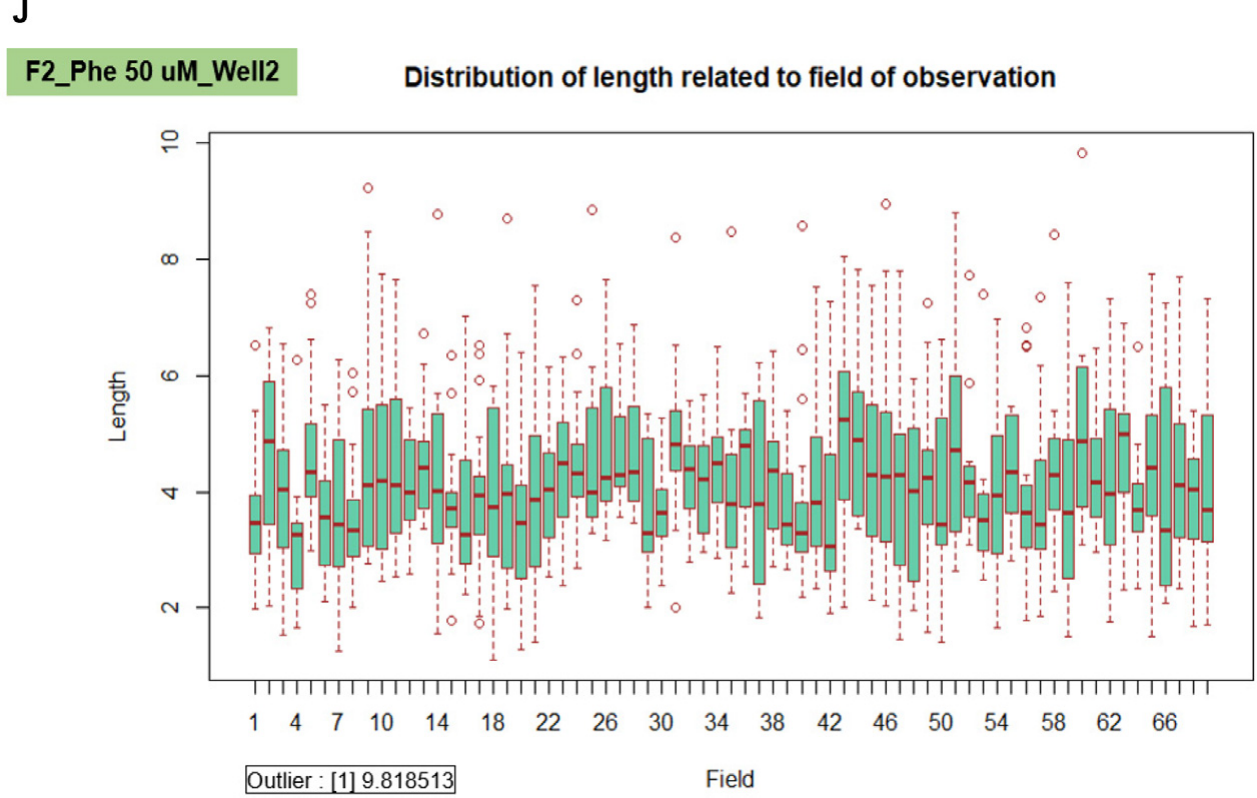
The validation of quality control in each well and field for the measurement of mitochondrial granularity and length levels. A. The well-related distribution of mitochondrial granularity levels in the duplicate wells of the 50 µM of phenanthroline treated cells and the untreated cells in individual fibroblast samples (F1, F2, and F3). B. The well-related distribution of mitochondrial length levels in the duplicate wells of the 50 µM of phenanthroline treated cells and the untreated cells in individual fibroblast samples (F1, F2, and F3). C. The distribution of mitochondrial granularity levels related to the field of observation in the conditions of the vehicle control (0 µM of phenanthroline) in well 1 of F1 fibroblasts. D. The distribution of mitochondrial granularity levels related to the field of observation in the conditions of the vehicle control (0 µM of phenanthroline) in well 2 of F1 fibroblasts. E. The distribution of mitochondrial granularity levels related to the field of observation in the conditions of the 50 µM of phenanthroline treated cells in well 1 of F1 fibroblasts. F. The distribution of mitochondrial granularity levels related to the field of observation in the conditions of the 50 µM of phenanthroline treated cells in well 2 of F1 fibroblasts. G. The distribution of mitochondrial length levels related to the field of observation in the conditions of the vehicle control (0 µM of phenanthroline) in well 1 of F2 fibroblasts. H. The distribution of mitochondrial length levels related to the field of observation in the conditions of the vehicle control (0 µM of phenanthroline) in well 2 of F2 fibroblasts. I. The distribution of mitochondrial length levels related to the field of observation in the conditions of the 50 µM of phenanthroline treated cells in well 1 of F2 fibroblasts. J. The distribution of mitochondrial length levels related to the field of observation in the conditions of the 50 µM of phenanthroline treated cells in well 2 of F2 fibroblasts.

We applied the granularity parameter from the CellProfiler program to determine the levels of mitochondrial fragmentation. From the texture analysis of the previous study, the granularity (granular spectrum) has been described as the size distribution normalized with integrated intensity [30]. Thus, the granularity parameter was used as a representative for the quantitative measurement of mitochondrial fragmentation. From the results, the percentage of mitochondrial fragmentation (granularity) from three different fibroblast cells (F1, F2, and F3) were significantly increased after 50 µM phenanthroline treatment compared with vehicle control (Fig. 17A). Conforming to the mitochondrial granularity, the average mitochondrial length substantially decreased in cells treated with phenanthroline at 50 µM when compared with vehicle control (Fig. 17B).We showed the distribution of mitochondrial granularity (Fig. 18A) and length (Fig. 18B) levels in duplicate wells in the condition of 50 µM of phenanthroline treated cells and untreated cells in individual fibroblast samples to account for any variations that might have an impact on the quality of the outcomes. The well-related distribution graph of mitochondrial granularity and length demonstrated a normal distribution with negligible variation between wells under all conditions (1 and 2). From the field-related distribution of mitochondrial granularity of observation, the graph in the conditions of F1_phe 0 µM_Well 1 (Fig. 18C), F1_phe 0 µM_Well 2 (Fig. 18D), F1_phe 50 µM_Well 1 (Fig. 18E), and F1_phe 50 µM_Well 2 (Fig. 18F) revealed a minor variation between the fields, as well as field-related the distribution of mitochondrial length, F2_phe 0 µM_Well 1 (Fig. 18G), F2_phe 0 µM_Well 2 (Fig. 18H), F2_phe 50 µM_Well 1 (Fig. 18I), and F2_phe 50 µM_Well 2 (Fig. 18J). We additionally utilized the R program to identify the outlier in each well's data. We discovered that there was just one outlier in each condition in Fig.18C - J.

## Conclusions

Our study suggested a method for assessing the mitochondrial status, including ROS accumulation, MMP, and their morphology in fibroblast cells stained by specific-fluorescent dyes and observed with the High Content Imaging System. It was a powerful technology for high throughput detection providing faster and more reliable results than the conventional fluorescent microscope. We hope that our work will be useful for research studies involving mitochondrial-related pathological diseases in humans. Additionally, we believe that these methods can be applied to other types of cells.

## Appendix Supplementary materials

Image, application 1

## References

[1] Fischer F., Hamann A., Osiewacz H.D. (2012). Mitochondrial quality control: an integrated network of pathways. Trends Biochem. Sci..

[2] Murphy M.P. (2009). How mitochondria produce reactive oxygen species. Biochem. J..

[3] van der Bliek A.M., Shen Q., Kawajiri S. (2013). Mechanisms of mitochondrial fission and fusion. Cold Spring Harb. Perspect. Biol..

[4] Machiraju, P., et al., *SS-31 Reverses mitochondrial fragmentation in fibroblasts from patients with DCMA, a mitochondrial cardiomyopathy*. 2019. 10.3389/fcvm.2019.00167 PMC6873783 31803760

[5] Carpenter A.E. (2006). CellProfiler: image analysis software for identifying and quantifying cell phenotypes. Genome Biol..

[6] Kamentsky L. (2011). Improved structure, function and compatibility for CellProfiler: modular high-throughput image analysis software. Bioinformatics.

[7] McQuin C. (2018). CellProfiler 3.0: Next-generation image processing for biology. PLoS Biol..

[8] Dickinson B.C., Lin V.S., Chang C.J. (2013). Preparation and use of MitoPY1 for imaging hydrogen peroxide in mitochondria of live cells. Nat. Protoc..

[9] Singh S. (2014). Pipeline for illumination correction of images for high-throughput microscopy. J. Microsc..

[10] Lee S.C., Bajcsy P. (2006). Intensity correction of fluorescent confocal laser scanning microscope images by mean-weight filtering. J. Microsc..

[11] Sezgin M., Sankur B. (2004). Survey over image thresholding techniques and quantitative performance evaluation. J. Electronic Imaging.

[12] Dickinson B.C., Chang C.J. (2008). A targetable fluorescent probe for imaging hydrogen peroxide in the mitochondria of living cells. J. Am. Chem. Soc..

[13] Lippert A.R., Dickinson B.C., New E.J. (2015). Imaging mitochondrial hydrogen peroxide in living cells. Methods Mol. Biol..

[14] Escada-Rebelo S. (2020). Fluorescent probes for the detection of reactive oxygen species in human spermatozoa. Asian J. Androl..

[15] Beyer A.M. (2014). An acute rise in intraluminal pressure shifts the mediator of flow-mediated dilation from nitric oxide to hydrogen peroxide in human arterioles. Am. J. Physiol. Heart Circ. Physiol..

[16] Domondon M. (2020). Renal glomerular mitochondria function in salt-sensitive hypertension. Front. Physiol..

[17] Fernandes J. (2017). From the cover: manganese stimulates mitochondrial H2O2 production in SH-SY5Y human neuroblastoma cells over physiologic as well as toxicologic range. Toxicol. Sci..

[18] Zorova L.D. (2018). Mitochondrial membrane potential. Anal. Biochem..

[19] Johnson L.V., Summerhayes I.C., Chen L.B. (1982). Decreased uptake and retention of rhodamine 123 by mitochondria in feline sarcoma virus-transformed mink cells. Cell.

[20] Smiley S.T. (1991). Intracellular heterogeneity in mitochondrial membrane potentials revealed by a J-aggregate-forming lipophilic cation JC-1. Proc. Nat. Acad. Sci. U.S.A..

[21] Sakamuru S. (2012). Application of a homogenous membrane potential assay to assess mitochondrial function. Physiol. Genomics.

[22] Sakamuru S., Attene-Ramos M.S., Xia M. (2016). Mitochondrial membrane potential assay. Methods Mol. Biol..

[23] Ni H.M., Williams J.A., Ding W.X. (2015). Mitochondrial dynamics and mitochondrial quality control. Redox. Biol..

[24] Pham N.A. (2004). Altered mitochondrial structure and motion dynamics in living cells with energy metabolism defects revealed by real time microscope imaging. Microsc. Microanal..

[25] Amchenkova A.A. (1988). Coupling membranes as energy-transmitting cables. I. Filamentous mitochondria in fibroblasts and mitochondrial clusters in cardiomyocytes. J. Cell Biol..

[26] Lea P.J. (1994). Variations in mitochondrial ultrastructure and dynamics observed by high resolution scanning electron microscopy (HRSEM). Microsc. Res. Tech..

[27] Heytler P.G., Prichard W.W. (1962). A new class of uncoupling agents–carbonyl cyanide phenylhydrazones. Biochem. Biophys. Res. Commun..

[28] Brennan J.P. (2006). FCCP is cardioprotective at concentrations that cause mitochondrial oxidation without detectable depolarisation. Cardiovasc. Res..

[29] Park S.J. (2012). Mitochondrial fragmentation caused by phenanthroline promotes mitophagy. FEBS Lett..

[30] Ravkin, I. and V. Temov. *Bit representation techniques and image processing*.

