# Supplementary material for: Methods for mitochondrial health assessment by High Content Imaging System
Source: MethodsX. 2022 Apr 2;9:101685. doi: 10.1016/j.mex.2022.101685 (PMC9026914; doi:10.1016/j.mex.2022.101685)
Supplement: Supplementary file 1 [file mmc1.docx]

**SUPPLEMENTARY INFORMATION**

**The process for determining mitochondrial H_2_O_2_ by CellProfiler program**

**
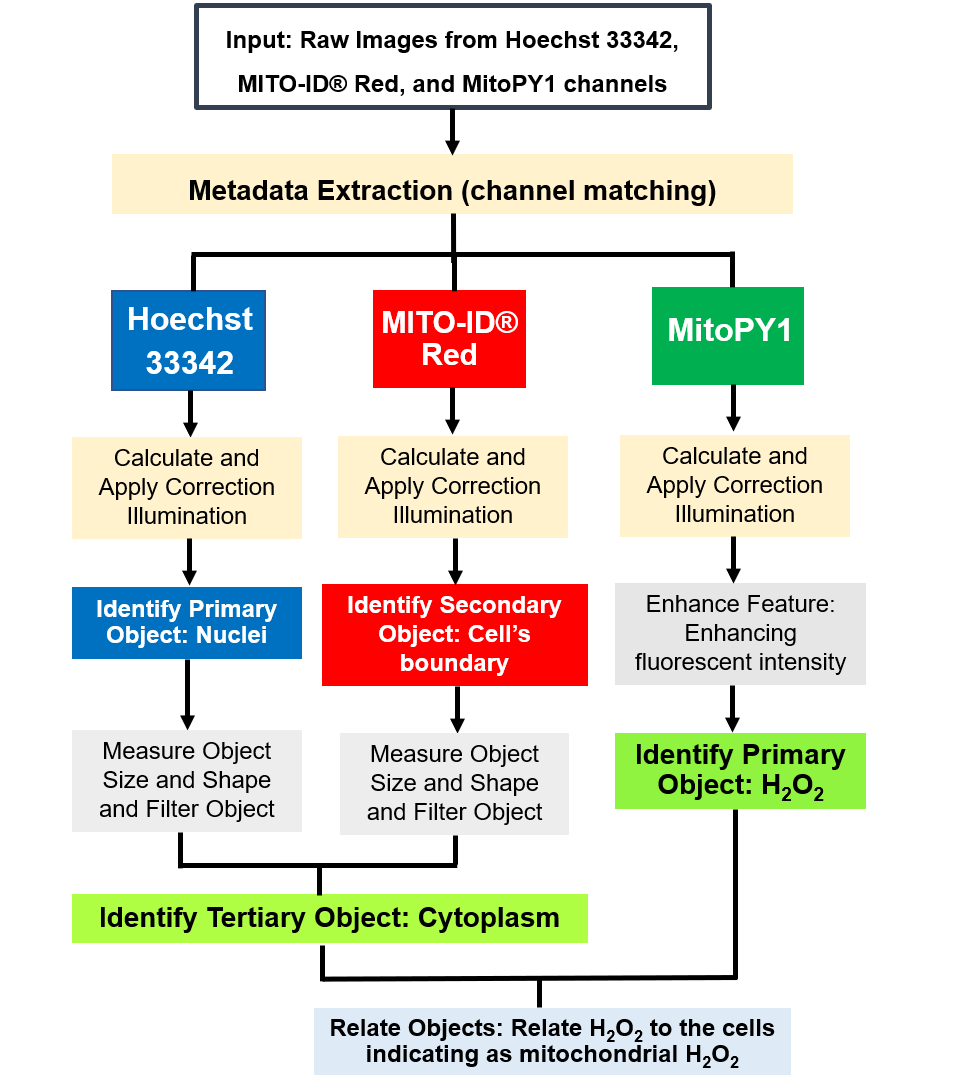
**

**Fig. S1.** The CellProfiler program's technique for calculating mitochondrial H_2_O_2_ is depicted in this diagram. The fluorescent raw images from the Operetta CLS were exported, and metadata was extracted from the file name to specify image information and match certain fluorescent channels. The Correction Illumination Calculate and Apply modules were used to apply the smoothed lightening pattern to each image. The principal object used to locate a cell was a stained nucleus dyed with Hoechst 33342 dye. The secondary object (a cell's boundary) was determined using the MITO-ID® Red channel. Objects with unsatisfactory main and secondary object characteristics were also filtered out using the Filter Object module. By subtracting the secondary object areas (whole-cell area) from the primary object areas, the cytoplasmic area was calculated (nuclear area). Using the Enhance Feature modules, the green-fluorescent intensity of the MitoPY1 channel was increased. The green-speckled fluorescence was then used to identify the H_2_O_2_. Using the Relate Object module to assess the H_2_O_2_ levels within mitochondria, the green-speckled fluorescence (H_2_O_2_) from MitoPY1 was assigned to relate with the cells observed from the Hoechst 33342 and MITO-ID® Red channels. The fraction of colocalized region of MitoPY1 and MITO-ID® Red fluorescent signals was used to calculate H_2_O_2_ levels within mitochondria.

**The process for determining MMP levels by the CellProfiler program**

**
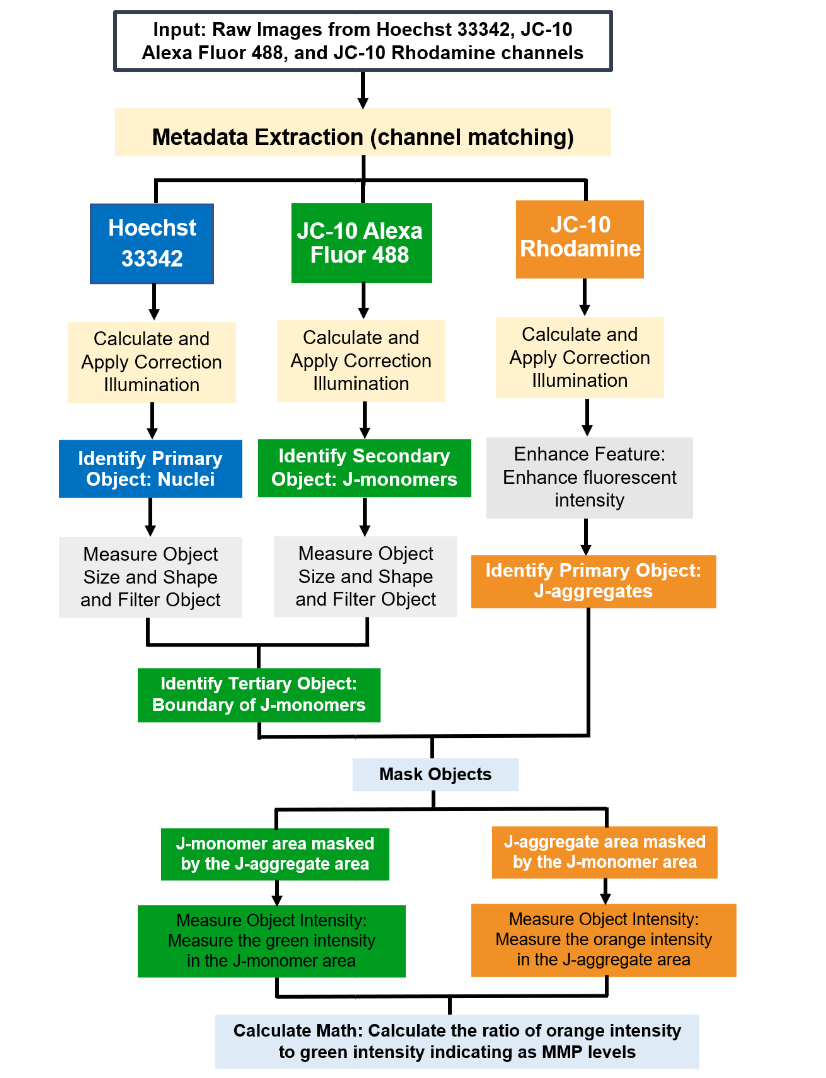
**

**Fig. S2.** The CellProfiler program's technique for assessing MMP levels is depicted in this diagram. The raw images of each fluorescent channel were exported from the Operetta CLS, and metadata was extracted from the file name to specify image information and match certain fluorescent channels. The smooth lightening pattern was applied to each image using the Correction Illumination Calculate and Apply modules. A stained nucleus colored with Hoechst 33342 dye was the principal object used to find a cell. The JC-10 Alexa Fluor 488 channel was used to identify the J-monomers (green-fluorescence). Objects with unsatisfied characteristics were also filtered out using the Filter Object module. The J-monomers were linked to nuclei and segmented using the Identify Tertiary Object modules. The J-aggregates (orange-fluorescence) were augmented by the Enhance Feature modules and specified by using the Identified Primary Object modules to determine the threshold of orange-fluorescence intensity in the JC-10 Rhodamine channel to sort out the polarized mitochondria. The Mask object module was used to keep overlapping regions and retain attempts to improve the specificity of the J-aggregate and J-monomer areas. The J-aggregate (orange-fluorescent region obscured by green region) and J-monomer intensities were calculated using the Measure Object Intensity module (green-fluorescent region masked by orange region). The fluorescent intensity ratio of J-aggregate to J-monomer was calculated using the Calculate Math module.

**The process for determining mitochondrial fragmentation and length levels by CellProfiler program**

**
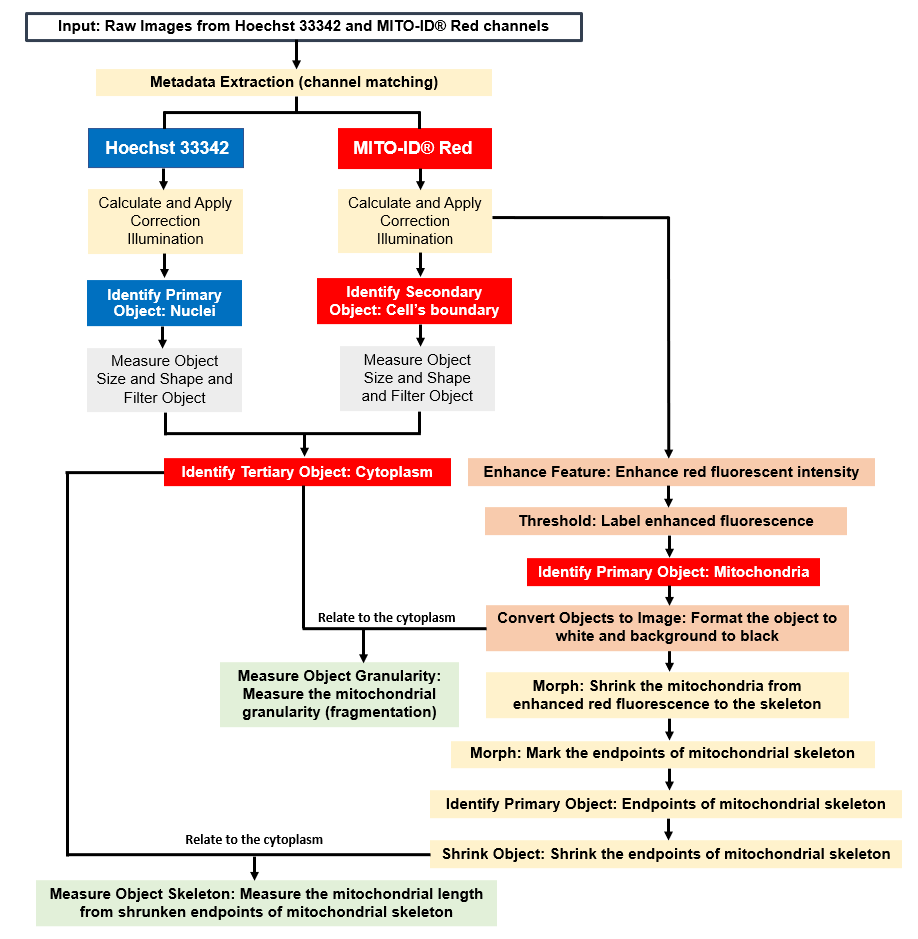
**

**Fig. S3.** The CellProfiler program's technique for determining the degrees of mitochondrial fragmentation and length levels is depicted in this diagram. The raw images from Operetta CLS fluorescent were exported, and metadata was extracted from the file name to specify image information and match specific fluorescent channels. The Correction Illumination Calculate and Apply modules were used to apply the smoothed lightening pattern to each image. A stained nucleus colored with Hoechst 33342 dye was the principal object used to find a cell. The MITO-ID® Red channel was utilized to determine the secondary object (a cell's boundary). Objects with unsatisfactory characteristics were filtered out using the Filter Object module. By subtracting the secondary object areas (whole-cell area) from the primary object areas (nuclear area), the cytoplasmic area was calculated. The fluorescence intensity of MITO-ID® Red channels was increased utilizing Enhance Feature modules to analyze the mitochondria. The Threshold module was used to further label the heightened red fluorescence. The mitochondria were then identified using the Identify Primary Object module. The Convert Objects to Image module was employed to format the object to white and the background to black. The Measure Object Granularity module was used to analyze the mitochondrial granularity from binary (white and black) color format of mitochondrial images that were associated to the cytoplasm. This module might detect mitochondrial fragmentation and provide a granular spectrum with various pixel sizes to reflect the percentages of fragmented mitochondria. The high degrees of granularity implied severely fragmented mitochondria. The Morph module was conducted to investigate mitochondrial network by reducing the mitochondria to a single line (skeleton) using the Skelpe method and utilizing the Endpoint method to detect the branch end of the mitochondrial skeleton. The Shrink Object module then shrank the mitochondrial skeleton endpoints. The distance between shrinking endpoints inside the mitochondrial skeleton which was related to the cytoplasm was measured using the Measure Object Skeleton.

**A**


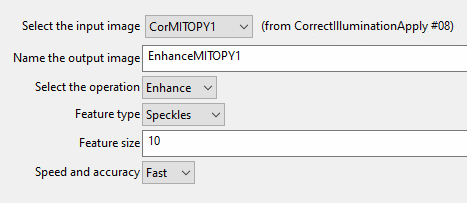


**B**


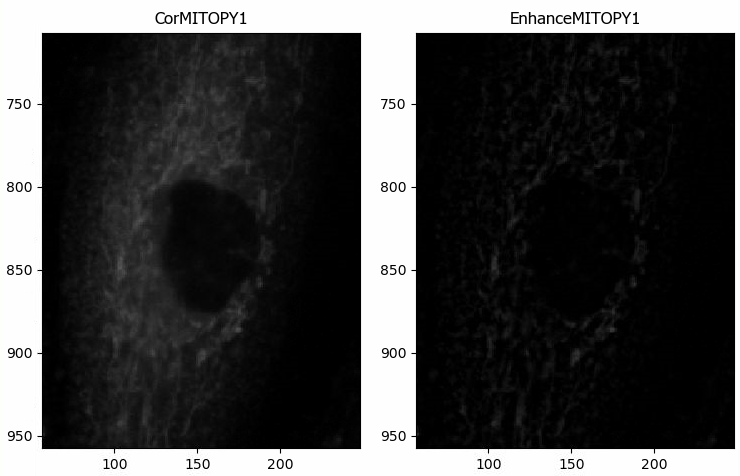

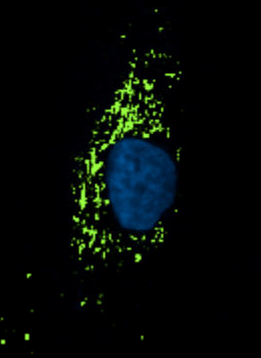


**10 µM**

**Fig. S4.** The Enhance Feature module‘s pipeline setting. The mitochondrial images applied Illumination correction were used as input images. The EnhanceMITOPY1 was the name given to the output images. In the MitoPY1 channel, the Enhance Feature module with the Speckle Feature type was used to heighten of larger intensity in contrast to its surroundings. The 10-feature size, or the diameter of the largest speckle, was set to determine the optimal filter size. The Fast algorithm was chosen to expedite the detection of speckles. B. The output image from the Enhance Feature module. Scale bars represent 10 µm.

**A**


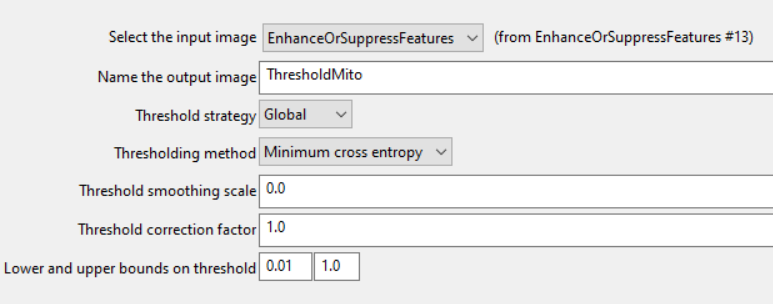


**B**


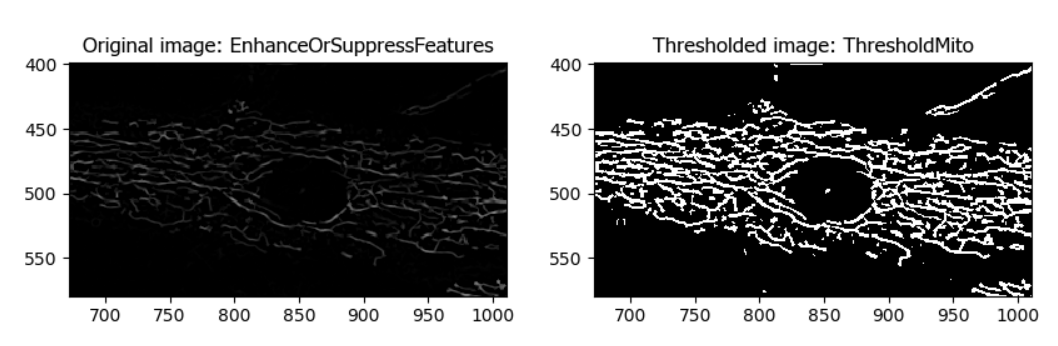


**Fig. S5.** The pipeline setting for the thresholding steps. A. The input images were the mitochondrial images that were enhanced the intensity from the Enhance or Suppress Feature modules. The output images were called the ThresholdMito. The Global Threshold strategy and the Minimum cross-entropy thresholding strategy were selected to classify foreground and background. These approaches use a single threshold value generated from the input image's unmasked pixels to identify pixels above and below the threshold as foreground and background, respectively. It's quick and reliable, especially if the background is consistent, e.g., after the images with the process of illumination correction. The Threshold smoothing scale, Threshold correction factor, and lower and upper bounds on threshold were set as 0.0, 1.0, and 0.01 to 1.0, respectively. B. The output image from the thresholding steps.
